# Childlessness and Development in Sub-Saharan Africa: Is There Evidence for a U-shaped Pattern?

**DOI:** 10.1007/s10680-022-09608-5

**Published:** 2022-03-10

**Authors:** Florianne C. J. Verkroost, Christiaan W. S. Monden

**Affiliations:** 1 Nuffield College and Department of Sociology, University of Oxford, Oxford, England; 2 Nuffield College, Department of Sociology and Leverhulme Centre for Demographic Science, University of Oxford, Oxford, England

**Keywords:** Childlessness, Development, Sub-Saharan Africa, Demographic and Health Surveys, Fertility

## Abstract

In high-income countries, women increasingly remain permanently childless. Little is known about the relationship between childlessness and socioeconomic development in non-Western societies and particularly sub-Saharan Africa. At lower levels of development, poverty-driven (i.e., involuntary) childlessness may decrease with increases in levels of development, while at higher levels of development opportunity-driven (i.e., voluntary and circumstantial) childlessness may rise with development. Thus, we expect a U-shaped relationship between childlessness and development overall. We examine this idea for sub-Saharan Africa. We further contribute by differentiating between female and male childlessness; and between involuntary, voluntary and circumstantial childlessness. Moreover, we construct new indicators of subnational historical development to assess both inter- and intra-country variation, and distinguish between three components (health, education and income) to investigate the drivers behind the hypothesized U-shaped relationship. Using 291 Demographic and Health Surveys between 1986 and 2018 from 38 countries and 384 regions, we find a U-shaped relationship between female childlessness and development, and a linear relationship for men. The U-shape for women results from negative associations of female involuntary childlessness with health and educational advancements, combined with positive correlations of voluntary and circumstantial childlessness with education and income improvements. While these positive associations are stronger among men than women, the negative relationships of involuntary childlessness with health and education observed for women are absent for men, resulting in an overall positive and linear relationship between development and childlessness among men. Our findings have implications for how we might expect childlessness rates to evolve with future levels of development.

**Supplementary Information:**

The online version contains supplementary material available at 10.1007/s10680-022-09608-5.

## Introduction

Substantial increases in permanent childlessness have gained growing interest from researchers and policy makers over the past decades. This interest has mainly been expressed for developed regions such as Europe and the USA (see for instance Frejka 2017; Kreyenfeld and Konietzka 2017). However, childlessness in less developed regions, and sub-Saharan Africa in particular, has hardly been investigated. One reason for this is that fertility rates have historically been high in sub-Saharan Africa, equal to almost seven births per woman on average in 1980 (The World Bank 2018). Although fertility rates have been decreasing in the region, they still equal almost twice the replacement level at an average of five births per woman in 2017. These high fertility rates have led to the assumption that childlessness is not significant in the region. While women in sub-Saharan Africa still have relatively many children on average, there are also a substantial number of women who never have children (Bongaarts and Casterline 2013; Inhorn and Patrizio 2015; Larsen 1994). This is particularly problematic as poverty may be the strongest driver of childlessness in sub-Saharan Africa (Baudin et al. 2020). Additionally, severe stigma and consequences are often attached to childlessness in the region, resulting in social exclusion, ostracism, divorce and abuse (Inhorn and Patrizio 2015).

To better understand the extent to which childlessness can be viewed as a response to structural socioeconomic conditions, we examine how childlessness varies across levels of socioeconomic development. In the early 1980s, Poston Jr. and colleagues (Poston and El-Badry 1987; Poston et al. 1985; Poston and Trent 1982) hypothesized that there is an association between development and the share of individuals who do not have children before the end of the (female) reproductive period. According to these researchers, the nature of this association changes with the level of development, resulting in a U-shaped association. This hypothesis has been investigated previously for the USA and several developing (of which 14 sub-Saharan African) countries (Baudin et al. 2020, 2015). However, in these studies different reasons for childlessness are predicted from an economic model based on a set of assumptions about childbearing conditions. Such indirect estimates may carry substantial uncertainty, which is why we deduce childlessness types from questions in the data directly. Furthermore, Baudin et al. (2020, 2015) use education as a proxy for development. However, we expect there to be heterogeneity in the distribution of development across its main components (Permanyer et al. 2015). More importantly, specific dimensions of development may influence various types of childlessness differently. For example, health might be more strongly related to involuntary childlessness while education might be more influential for voluntary childlessness (Poston and Trent 1982). Therefore, we consider development across its three domains of health, education and income. Finally, in the aforementioned studies (Baudin et al. 2020, 2015), the macro-level hypotheses are investigated on the national level. Nonetheless, levels of development vary significantly within countries, and particularly low- and middle-developed countries (Permanyer and Smits 2020). To ensure this variation is not lost, we investigate these hypotheses on the subnational level.

In this study, we aim to fill the gaps in the literature by answering the following three questions for sub-Saharan Africa. First, how are permanent childlessness and indicators of development at the subnational level related? Second, how does this association differ across types of childlessness (i.e., involuntary, voluntary and circumstantial childlessness)? Third, are there gender differences with respect to the first two questions? We answer these questions by using hierarchical models in conjunction with data from 291 Demographic and Health Surveys (DHS) collected between 1986 and 2018 throughout 38 sub-Saharan African countries and 384 subnational regions. We exploit various other data sets to construct innovative historical subnational-level development indicators and take into account other factors that may be related to childlessness, such as HIV prevalence and marriage market sex ratios. We define permanent childlessness as the percentage of individuals in a region-year combination that “have never born any children before reaching age 40”. This means that all infertile individuals, even those who have adopted children or have stepchildren, are classified as childless and that individuals whose only child or children died young are not defined as childless. In our framework, involuntary childlessness is due to infertility,^1^ voluntary childlessness refers to the choice not to have children, and circumstantial childlessness is a consequence of not having (had) a partner to have children with.

The contribution of our study is fourfold. First, we provide a comprehensive overview of childlessness levels across sub-Saharan Africa and how these levels relate to development indicators. Previous research has shed light on the relationship between development and childlessness at lower levels of development by examining a limited selection of low-income countries in Asia, Central and Latin America and sub-Saharan Africa. These studies found support for the hypothesis that development is negatively related with involuntary childlessness and positively related with voluntary childlessness (Baudin et al. 2020; Poston and El-Badry 1987; Poston et al. 1985; Poston and Trent 1982). However, to date very little is known about the association between childlessness and development across the whole of sub-Saharan Africa, a region that has seen substantial improvements in development over the last decades. In this study, we shed more light on this association.

Second, by exploiting comparative data with large sample sizes, we can distinguish between involuntary, voluntary and circumstantial childlessness in the relationship with development. We may expect the association with development to be negative for involuntary childlessness due to reductions in poverty and poor health, but positive for voluntary and circumstantial childlessness due to economic and educational expansion, individualization and changing norms on family formation, resulting in a U-shaped relationship between overall levels of childlessness and development (Baudin et al. 2020; Poston et al. 1983). To understand the drivers and mechanisms behind the potentially U-shaped relationship between overall childlessness and development, it is crucial to distinguish between different types of childlessness. However, hardly anything is currently known about the association between development and different types of childlessness in sub-Saharan Africa, as the main focus has always been on infertility (e.g., Casterline and Han 2017; Inhorn and Patrizio 2015; Larsen 2000, 2003; Mascarenhas et al. 2012; Rutstein and Shah 2004). Poston et al. (1983) have tried to distinguish between voluntary and involuntary female childlessness in Lesotho and Kenya, but their samples were too small to draw robust conclusions. Baudin et al. (2020) have also aimed to distinguish between poverty- and opportunity-driven childlessness, but by predicting these indicators from economic models rather than estimating them from available data directly. By constructing a decision tree and exploiting the richness of the DHS, we are able to examine different types of childlessness besides infertility.

Third, we construct completely new historical indicators for development components at the subnational-level. This allows us to study the relationship between childlessness and development across a wider range of development than ever before. Not only does the distribution of development vary across its components (i.e., education, health and income), there is also substantial variation in development levels between and within countries (Permanyer et al. 2015). These sources of heterogeneity in development occur particularly in low- and middle-developed countries (Permanyer and Smits 2020), and this variation is lost in most macro-level studies. By harmonizing subnational information in our data and recovering historical indices of development at the subnational level, we can examine not only inter- but also intra-country variation in childlessness and development.

Fourth, we analyze women and men separately to examine whether gender differences exist in the relationships between types of childlessness and indicators of development. For example, (female) educational and occupational attainment as well as autonomy increases with higher levels of development (World Economic Forum 2018). As a result, voluntary childlessness may become more attractive for women in particular because of the increased opportunity cost of having children (Becker 1960; Neyer et al. 2017). On the other hand, men may increasingly remain childless by circumstance as a consequence of being left behind on largely hypergamous mating markets (Kreyenfeld and Konietzka 2017). Very little is currently known about male childlessness in sub-Saharan Africa, as female surveys have been collected for a longer period of time and childlessness research has usually focused on females (Dyer et al. 2004). We aim to fill this gap by including men in our analyses.

## U-shape of Childlessness and Development

In this section, we discuss mechanisms at play in the relationship between (different indicators of) development and (different types of) childlessness. Some of these mechanisms concern factors that affect childlessness directly; others may have indirect effects as intentions for a smaller family size may lead to childlessness through childbirth postponement (Burkimsher and Zeman 2017; Madsen et al. 2017).

At the lowest levels of development, childlessness is mainly caused by poverty-driven infertility (Baudin et al. 2020; Poston and Trent 1982). Although fertility rates have generally been high in sub-Saharan Africa, infertility has also been prevalent (Bongaarts and Casterline 2013). Infertility or involuntary childlessness may derive from for example malnutrition, famine, disease (mainly sexually transmitted or pelvic inflammatory), female circumcision, exposure to chemicals and radiation or a lack of good pre-, ante- and postnatal care (in the case of secondary infertility) (Larsen 1994; Poston et al. 1983). Increases in development in terms of nutrition as well as medical expertise and expenditure reduce the prevalence of poverty-driven infertility through better prevention and treatment of diseases and other health problems (Inhorn and Patrizio 2015). With growing medical expertise and expenditure, we also expect to see increasing usage of medically assisted reproduction (MAR), which will reduce infertility rates directly, and also indirectly by assuring positive birth outcomes despite postponement of childbearing (Marmot 2005; Casterline and Han 2017). Although the influence of MAR on childlessness levels may still be negligible in sub-Saharan Africa, given that these technologies have remained largely inaccessible throughout the region (Inhorn and Patrizio 2015), it may be of increasing relevance for some of the most highly developed areas in our study.

At higher levels of development, especially driven by educational attainment and economic well-being, we might expect a larger contribution of voluntary and circumstantial childlessness to overall levels of childlessness (Poston and Trent 1982). Sub-Saharan Africa has seen an increase in overall gender equality, also in terms of economic opportunity and educational attainment (World Economic Forum 2018). These improvements are expected to be positively related to childlessness through two mechanisms. First, according to the ideas of Becker (1981), the quality of children is larger at higher levels of economic well-being, which results in a lower demand for children. Lower fertility intentions may result in childbirth postponement, which may in turn lead to age-related infertility or the choice to forego having children (Burkimsher and Zeman 2017; Madsen et al. 2017). Second, higher levels of development come with higher levels of female educational and occupational attainment. On the one hand, this phenomenon drives voluntary childlessness particularly among women through increased opportunity cost of having children (Neyer et al. 2017), higher levels of autonomy and self-sustenance (Oppenheimer 1994) and more diverse life opportunities (Rindfuss and Bumpass 1976; Surkyn and Lesthaeghe 2004; van de Kaa 1996). On the other hand, this process may increasingly leave men childless by circumstance, as they are left behind on the mating market (Kreyenfeld and Konietzka 2017). That is, women tend to marry men with similar (homogamy) or higher (hypergamy) levels of education while men tend to marry women who are at most as highly educated as themselves (hypogamy), which may lead to an imbalance in the marriage market (van Bavel 2012; Chudnovskaya 2019). Although this mechanism may not be commonly in place in sub-Saharan Africa (yet), it could play a role in some of the most highly developed urban areas.

Further, higher levels of development, particularly through educational expansion, bring exposure to global culture, which may affect cultural norms and values about family formation and childbearing. In sub-Saharan Africa, there is a strong desire for children and childbearing has been nearly universal (Bongaarts and Casterline 2013). There is a strong stigma associated to remaining childless, often resulting in social exclusion, divorce and abuse (Inhorn and Patrizio 2015). Nevertheless, with increases in development sub-Saharan Africa has also seen increases in ages at first marriage (Shapiro and Gebreselassie 2014), divorce (Clark and Brauner-Otto 2015), fertility postponement (Moultrie et al. 2012), contraceptive use (Tsui et al. 2017) and cohabitation (Odimegwu et al. 2018) and a decline in the total fertility rate (Bongaarts and Casterline 2013). Although this does not necessarily mean that we should expect patterns of fertility and family behavior to simply follow a path of convergence to patterns observed in high-income countries (Pesando and GFC team 2018; Thornton 2013), these observed transitions may come with increased desires for feminism, autonomy and individualism. As a consequence, the concept of “childfree” lives may emerge (Beaujouan et al. 2016; Letherby 2002), whereby desires for recreational and luxurious opportunities can be met without the burden of children (van Bavel and Kok 2010). This may also lead to postponement in marriage and childbearing, increasing the share of people foregoing having children (Burkimsher and Zeman 2017; Madsen et al. 2017). With such shifts in cultural values, voluntary and circumstantial childlessness may become not only more prevalent but also more socially accepted (van Balen and Bos 2009; Burkimsher 2014; van Balen 2008).

**Fig. 1:**
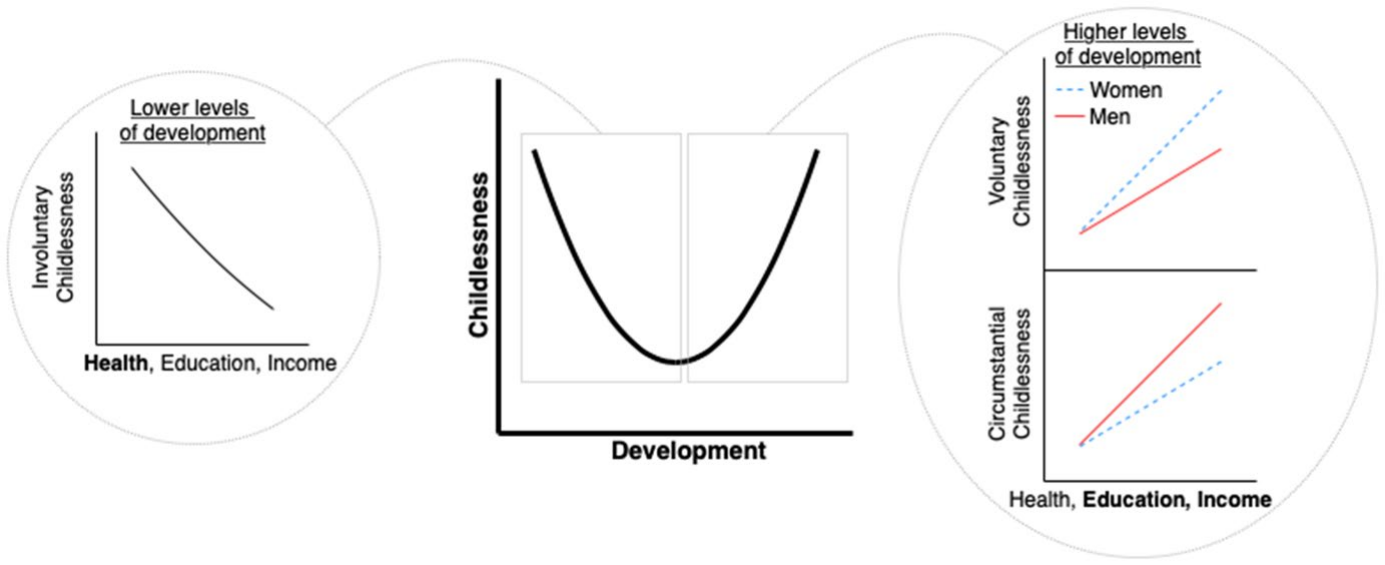
Overview of hypotheses regarding the relationship between childlessness types and components of development

Summarizing, we might expect that involuntary childlessness is negatively associated with development, and voluntary and circumstantial childlessness are positively associated with development (Poston and Trent 1982). Involuntary childlessness is expected to contribute most strongly to overall childlessness levels at lower levels of development, while at higher levels of development voluntary and circumstantial childlessness are expected to drive overall childlessness levels (Baudin et al. 2020). Therefore, we hypothesize that childlessness declines with development at lower levels of development and increases with development at higher levels of development, resulting in a U-shaped relationship between childlessness and development (see Fig. 1). Table 1 sums up expectations and mechanisms for the three different components of development by childlessness type. In our analyses, we first investigate whether there is indeed a U-shape between overall levels of childlessness and overall levels of development, and then split childlessness into its different types (i.e., involuntary, voluntary and circumstantial) and development into its different components (i.e., health, education and income) to identify potential mechanisms operating behind this U-shape.

**Table 1:** Overview of hypotheses regarding the relationship between childlessness types and indicators of development

Childlessness type	Development indicator	Relationship	Development level	Mechanism(s)	Gender difference
Involuntary	**Health**	Negative	Lower	Better prevention and treatment of disease; more access to medically assisted reproductive technologies; more reassurance of positive birth outcomes	None
	Education			Indirect: more educational expertise and skills improve medical technologies and health(care), in turn increasing fertility	
	Income			Indirect: higher income improves nutrition and expenditure on health-relevant products, in turn increasing fertility	
Voluntary	Health	Positive	Higher	More control over (postponing) childbearing through contraception; lower child mortality and thus desire for children	Stronger for women
	**Education**			Changing family values; marriage and childbirth postponement; increased opportunity cost of children; higher quality of and thus lower demand for children; more diverse life opportunities besides family care	
	**Income**				
Circumstantial	Health	Positive	Higher	Less distorted marriage market sex ratios as consequence of epidemics (particularly HIV/AIDS)	Stronger for men
	**Education**			Changing family values; marriage and childbirth postponement; more competition among women for higher-educated men	
	**Income**				

We need to consider two phenomena in our analyses that are particularly relevant to sub-Saharan Africa: polygyny and HIV. Although the prevalences of polygyny and HIV are not homogeneous across the region and both have started to decrease over the past years (Fenske 2015; Steinbrook 2008), they may still be relevant for childlessness. Polygyny prevalence and overall childlessness may be expected to be negatively associated, because polygyny has been shown to increase both aggregate and individual fertility via fertility contagion and rising marriage rates (Cahu et al. 2014). The influence of polygyny prevalence on childlessness may be different for men and women, however. On the one hand, polygyny may decrease male childlessness through increased fertility via higher chances of pregnancy with multiple wives (Cahu et al. 2014; Musham 1956; Rossi 2019; Schoumaker 2019). On the other hand, female childlessness is likely higher in regions where polygyny is high, mainly because of lower frequency of sexual intercourse, higher chances of venereal diseases and the selection of infertile women into polygynous unions (i.e., female infertility incentivizes husbands to practice polygyny) (Cahu et al. 2014; Lardoux and van de Walle 2003; Musham 1956). Further, we may expect that higher HIV prevalence increases childlessness and reduces fertility, especially in the presence of other sexually transmitted diseases (van Leeuwen et al. 2006). As HIV is more prevalent among (young) women in the region and may lead to AIDS and eventually death when left untreated (Hegdahl et al. 2016), HIV epidemics may lead to shortages of women on the marriage market and potentially a larger share of men remaining unmarried and childless.

## Data

We use the Demographic and Health Surveys (DHS) Program data (ICF International, 1986-2018). Note that we do not make use of the World Fertility Surveys as their sample sizes are too small for the purposes of our subnational-level analyses. The main advantages of the DHS for our purpose are the large sample sizes; the availability of both male and female surveys; and the possibility to assess inter- and intra-country differences in childlessness. As only a relatively small percentage of the population will remain permanently childless, large sample sizes are necessary to be able to draw reliable conclusions for this small group of the population. Comparability across countries and subnational regions provides us with the necessary variability in development needed for our analyses and also allows us to study differences across and within countries in the associations between types of childlessness and development.

We use data from all DHS surveys for sub-Saharan Africa that have information on childbirth and marital status as well as sufficient information on subnational regions to harmonize these subnational regions over time. We do not consider surveys that only interviewed ever-married women and men. A total of 291 individual-level (169 female and 122 male) surveys conducted between 1986 and 2018 throughout 38 sub-Saharan African countries and 384 subnational regions met these criteria. These 291 surveys are comprised of 255 standard DHS surveys, 27 Malaria Indicators Surveys (MIS) and 9 AIDS Indicator Surveys (AIS). A list of countries with sample sizes before and after data operationalization is included in Table S1 in Supplementary Information (SI) A. More information on all DHS surveys in our analysis can be found in Figure S1 in SI B.

### Aggregation

We aggregate the individual-level data in the DHS surveys to country- and subnational region-level data. Our main units of analysis are region-year combinations. We select individuals aged 40 and older because we aim to study permanent childlessness. The upper age limit for interviews is 49 for most female surveys and 59 for men. Two female and nine male surveys interviewed respondents up to age 64.^2^ While some men and women will have their first child after age 40, this group is very small (see Figure S2 in SI C). Although a cutoff at age 40 might slightly overestimate childlessness, setting a higher cutoff age (e.g., 45) would limit the sample size too much and result in inaccurate estimates. Using the lower bound of 40 for both women and men therefore allows us to study permanent childlessness while maximizing our sample size.

Our individual-level sample includes 269,514 women and 136,748 men. We have 1,749 subnational region-year combinations for women with an average sample size of 154 women. Each subnational region on average has 4.6 observations (i.e., years) ranging between 1986 and 2018. The rounded average most recent year of observation is 2014. For men, there are 1,213 subnational region-year combinations with an average sample size of 113 men. The average number of observations (i.e., years) per subnational region is 3.3. The years of observations vary between 1991 and 2018. The average most recent year of observation is 2013. Figure S6 in SI G shows childlessness levels by sample size. Table 2 shows the descriptive statistics for our sample.

In the upcoming sections, it is important to keep in mind that indicators for development as well as HIV prevalence and marriage market sex balance are lagged to reflect the year in which the average age of the respondents in the region-year combination would have been 19 for women and 24 for men. These factors mostly play a role for childlessness during partnership formation and the early childbearing ages, and not so much after age 40 when the respondents are interviewed. We choose ages 19 for women and 24 for men, because these are the median childbearing ages in our samples, corresponding to the numbers found by Dunlop et al. (2018); Westoff (2003).

**Table 2:** Descriptive statistics for subnational regional-level data

	Men				Women			
	Mean	St. Dev.	Min	Max	Mean	St. Dev.	Min	Max
Number of individuals	112.735	118.264	2	1552	154.096	166.659	1	2950
Average age at first birth	26.827	2.546	20.943	60	23.042	7.627	15.182	43.250
Average age at first marriage/cohabitation	25.546	2.308	19.250	37	18.808	2.260	12.286	27.826
Average age at first sex	19.878	2.187	14.699	28.077	16.962	1.408	12.833	22.091
HIV prevalence (%)	0.652	1.138	0	11.2	2.288	3.398	0	28.4
Number of women per 100 men	100.508	3.827	89.968	117.813	100.182	3.434	89.107	120.045
Urban residence (%)	31.318	24.761	0	100	30.973	24.667	0	100
Polygyny prevalence (%)	21.229	15.169	0	100	34.225	19.887	0	100
Year	2005.655	7.379	1991	2018	2006.170	8.243	1986	2018
Average age	47.440	2.334	41	59.567	43.987	0.853	40	51.135
**SHIHD**								
Overall	0.169	0.063	0.049	0.420	0.179	0.060	0.052	0.433
Education	0.107	0.070	0.006	0.366	0.122	0.073	0.006	0.409
Health	0.143	0.042	0.044	0.284	0.149	0.040	0.032	0.295
Income	0.362	0.087	0.188	0.775	0.357	0.084	0.168	0.780
**Childlessness**								
Overall (%)	3.499	3.734	0	32.653	2.860	2.650	0	20.690
Voluntary (%)	0.300	1.086	0	18.367	0.127	0.434	0	5
Involuntary (%)	1.090	1.707	0	20	1.490	1.890	0	20.690
Circumstantial (%)	2.067	2.665	0	17.647	0.628	1.163	0	11.454
**Marital Status**								
Married/cohabiting/relationship (%)	90.876	7.565	47.059	100	78.996	12.953	0	100
Never married (%)	2.492	4.430	0	39.286	3.206	10.404	0	100
Divorced/separated (%)	4.767	4.495	0	40	8.693	6.006	0	36.957
Widowed (%)	1.865	2.473	0	20	9.106	6.259	0	34.127
Number of subnational regions	372				384			
Number of subnational region-year combinations	1,213				1,749			

### Childlessness

Our dependent variable represents the percentage of childless individuals aged 40+ in a particular subnational region-year combination. This variable has been constructed by aggregating individual-level dichotomous childlessness based on the number of total children ever born (i.e., “1” if the total number of children equals zero and “0” if this number is larger than or equal to one). Stillbirths, miscarriages and other forms of terminated pregnancies are not included as live births. We do not take into account adopted and stepchildren.

**Fig. 2:**
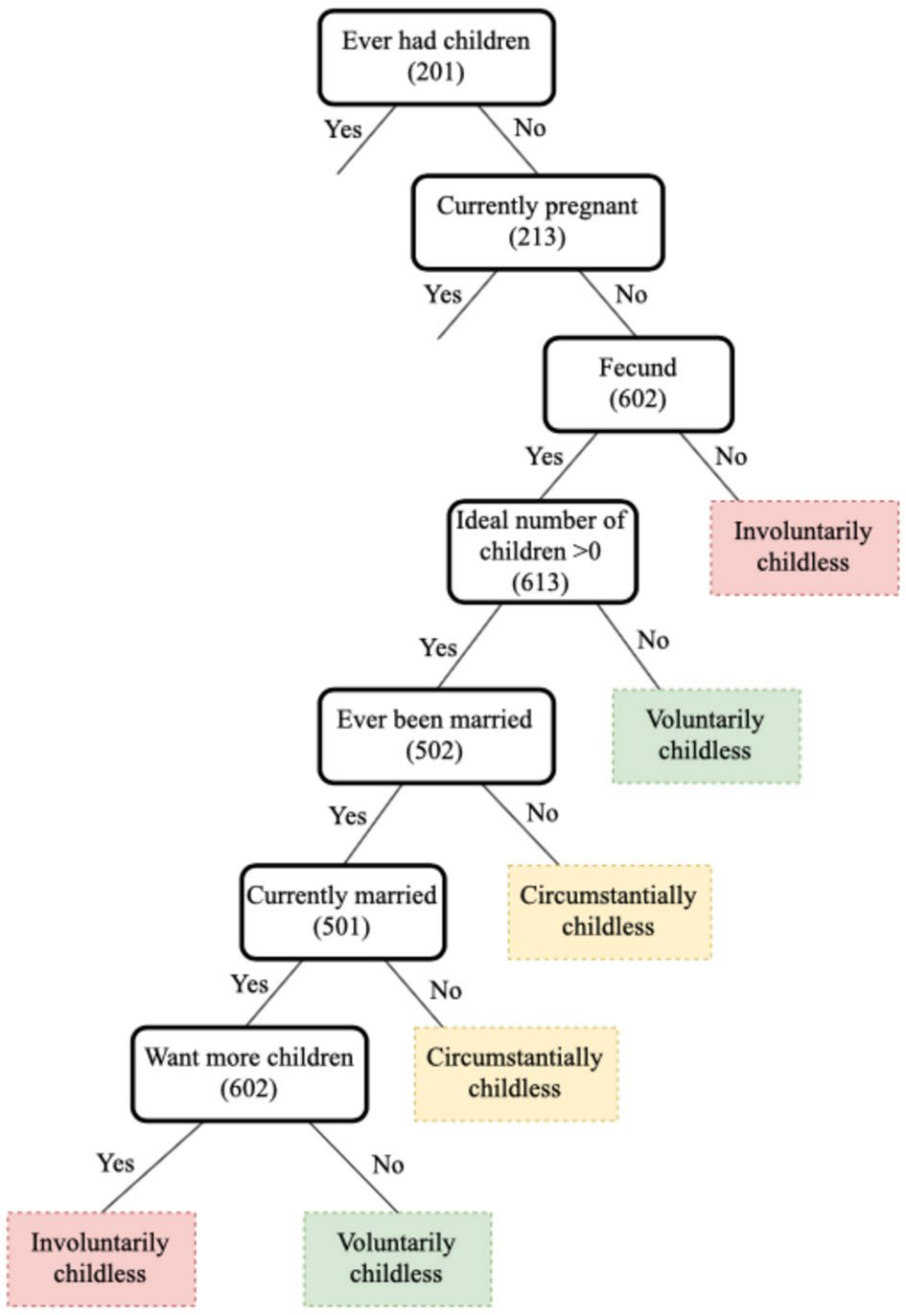
Decision tree for the classification of types of childlessness (DHS question number between brackets)

Three different variables represent the percentage of involuntarily, voluntarily and circumstantially childless individuals in a particular subnational region-year combination. These variables have been aggregated from an individual-level categorical variable that has been constructed on the basis of the possible combinations of responses to three questions, namely i) a question that asks about fertility preferences; ii) a question regarding the ideal number of children one would have had if they could turn back time and irrespective of fertility outcomes; and iii) marital status. Figure 2 shows a decision tree for this classification. If respondents aged 40+ have never had any children and are not pregnant at the time of interview, they are classified as childless. If these childless respondents report that they are not fecund (i.e., they/their partner cannot get pregnant regardless of whether they desire to have children), or report that they are fecund, want to have children and are currently married but still have not been able to get pregnant, they are classified as involuntarily childless. Voluntarily childless adults are those who do not want to have any children. Those who are fecund, want to have children, but are currently not married (either because of never having been married or because of having been separated, divorced or widowed) are classified as circumstantially childless. Respondents who do not know whether they want to have children and what their ideal number of children is, have been classified as undecided. We do not make inferences for this group because they would be uninformative and the group is relatively small.

### Development

We construct our main independent variable by combining the national-level Historical Index of Human Development (HIHD) (Prados de la Escosura 2015a, b) with the non-historical Subnational Human Development Index (SHDI) (Smits and Permanyer 2019) to create our own Subnational Historical Index of Human Development (SHIHD). The HIHD is a cross-country comparative indicator and a composite measure of life expectancy at birth, educational enrollment rates, adult literacy and gross domestic product per capita. The HIHD indicators are available approximately every five years in the period relevant to us and we linearly interpolate the years in-between. The SHDI is constructed in the same way as the Human Development Index (HDI) (UNDP 2015), namely from life expectancy at birth, expected years of schooling, average years of schooling and gross national income per capita, and coincides with the HDI on the national level. We further distinguish between the three main components of development, namely education, income and health, which are separately available for both the HIHD and SHDI. Both the HIHD and SHDI components are measured on a zero-to-one scale.

For the overall SHIHD as well as its three components, we use the national-to-subnational ratios of the earliest available year (usually 1990) of the SHDI (component), and then we use these ratios in combination with the national-level HIHD (component) to obtain subnational development indicators for years before 1990. For example, if we want to construct the SHIHD for the Atacora region in Benin in 1950 and the SHDI for Benin in 1990 is 0.348 overall and 0.304 for the Atacora region, and the HIHD for Benin is 0.055 in 1950, then we construct the 1950 SHIHD for the Atacora region as $0.304/0.348 \cdot 0.055=0.048$. We thus assume that during the years leading up to 1990, the ratios of subnational and national development are homogeneous over time. The country-specific trends of national and subnational SHDI components in SI E show that this is a reasonable assumption for both indicators as these ratios are generally stable over the period for which we do have data (1990-2017). Finally, for every subnational region-year combination, we use the SHIHD indicator for the year in which the average age of the respondents in that subnational region was 19 (women) or 24 (men), as aforementioned. For example, the Rift Valley subnational region in Kenya in 2014 had 2,950 female respondents aged at least 40 with an average age of 43.8. For this subnational region-year combination, the relevant development indicator would come from year $(2014 - (43.8 - 19 )) \approx 1989$.

To obtain a better understanding of the substantive meaning of the SHIHD, it is important to note that our SHIHD indicator as well as its components (education, income and health) follow the scale of the HIHD (Prados de la Escosura 2015a, b), which is based on the UNDP’s version of the HDI (UNDP 2015). Following Prados de la Escosura (p. 227–228, 2015b), we use the same bounds as the UNDP but apply a nonlinear rather than linear transformation to the 0-1 scale, as explained below. Health is measured in terms of life expectancy at birth, i.e., the average number of years alive remaining for individuals reaching the specified ages if they were subjected to the mortality rates corresponding to the year(s) these life expectancies relate to. Therefore, from here onwards we will refer to the health component as “life expectancy”. Education is a composite measure (i.e., unweighted geometric average) of the adult literacy rate (i.e., the share of the population aged 15+ who are able to write and read) and gross enrollment rate (i.e., the share of the population aged 5–24 enrolled in primary, secondary or tertiary education). Income is expressed in terms of GDP per capita, measured by 1990 dollars corrected for its purchasing-power (i.e., the difference in cross-country price levels, Geary-Khamis 1990 $). The three measures are nonlinearly transformed to a zero-to-one scale for comparability across countries and indices. Life expectancy and education are transformed as

1 $$\begin{aligned} \text {Life expectancy} = \frac{\ln {(85 - 20)}-\ln {(85 - \text {Life expectancy})}}{\ln {(85 - 20)}} \end{aligned}$$

and

2 $$\begin{aligned} \text {Education} = \frac{\ln {(100 - 0)}-\ln {(100 - \text {Education})}}{\ln {(100 - 0)}} \end{aligned}$$

in order to take into account that increases of the same absolute size are more significant at higher than lower levels of the index. Income is log-transformed by

3 $$\begin{aligned} \text {Income} = \frac{\ln {(\text {Income})} - \ln {(100)}}{\ln {(46949)} - \ln {(100)}} \end{aligned}$$

to ensure returns decline as the level of the index increases. The overall HIHD is then computed as the geometric mean of these three transformed indices. The lower and upper bounds for each indicator (“goalposts,” e.g., 20 and 85 for life expectancy) are similar to those used to compute the HDI (United Nations Development Programme [UNDP] 2019). Note, however, that because of the log transformations of the SHIHD components, life expectancy and education are well-defined in the [0, 84] and [0, 99] ranges, respectively. While this means the indicators are ill-defined beyond 84, respectively, 99, this does not pose any problems for our study as these upper bounds are not reached anywhere in sub-Saharan Africa. Substantively, a high score (close to one) on one of the indices means that the value of this indicator is close to the upper bound (e.g., a life expectancy score close to one indicates life expectancy close to 84 years), whereas a low score (close to zero) indicates proximity to the lower bound (e.g., an income score close to zero indicates GDP per capita in G-K 1990 dollars close to $$\log {100}$$ $). To illustrate, the 2015 EU-28^3^ and sub-Saharan African averages on the indicators were 0.74, respectively, 0.27 for the overall HIHD; 0.80 resp. 0.23 for education; 0.83 resp. 0.42 for income and 0.62 resp. 0.23 for life expectancy. Further, to obtain a sense of how the HIHD and its components have developed since 1938 in sub-Saharan Africa, Figure S5 in SI F shows the trends of the HIHD and its components per country.

### Other Factors

The percentage of individuals living in an urban area is measured by the de facto place of residence (rather than de jure place of residence, see Caselli et al. (2005)) and is calculated for each subnational region-year combination.

Marital status is reflected by the percentage of individuals who are i) never married; ii) married, in a relationship or cohabiting; iii) divorced or separated; or iv) widowed for a particular subnational region-year combination. Four variables are constructed on the basis of responses to questions about current and former marital status.

As an indicator for marriage market sex balance, we use the number of women per 100 men in the age group between 15 and 24. We choose this age group because in our data the median ages at which most women and men marry are 17 and 23, respectively, which also corresponds with findings by Marston et al. (2009). This variable is available only on the national level because of a lack of age- and sex-specific population data at the subnational level. We use the sex-specific 2017 World Population Prospects by the United Nations Desa/Population Division (United Nations Desa/Population Division 2017a, b) for the year when the average age of all individuals in the subnational region would have been 19 (women) or 24 (men) to approximate the sex balance at the age at which childbearing usually takes place in sub-Saharan Africa.

Polygyny is represented as the percentages of men and women in a particular subnational region-year combination who, respectively, report having multiple wives or their partner having at least one other wife. As there seems to be more uncertainty about polygyny in the female data, likely because women may not always be aware of their husband having multiple wives, we impute polygyny prevalence for region-year combinations from the male data when missing or mostly uncertain in the female data. Respondents who are not in a relationship are classified as being monogynous. There are seven surveys for which the polygyny variable only consists of “not applicable” responses, of which five are standard DHS and 27 are MIS. No further information is given by the data collectors as to why the polygyny information is missing in the standard DHS samples. For all 27 MIS, the polygyny question has not been asked.

We include three indicators to have an idea of family postponement: average ages at first sex, first marriage and first childbirth. Postponement may play an important role in childlessness. We use the average first ages for all individuals in each particular subnational region and year. We use the age at first cohabitation as a proxy for the age at first marriage for surveys where the latter was not asked.

Sex-specific HIV prevalence indicators are added for every subnational region-year combination. As most individuals in sub-Saharan Africa make childbearing decisions around the age of 20, we use HIV prevalence indicators for individuals aged between 15 and 24 and we match these to the year when the average age of the respondents in the subnational region would have been 19 (women) or 24 (men). We obtain national-level data from the Joint United Nations Programme on HIV/AIDS (UNAIDS) (2017a) and subnational-level data for different sets of countries from the US Agency for International Development (2018) and Joint United Nations Programme on HIV/AIDS (UNAIDS) (2017b). As national-level data are available from 1990 to 2017 for the majority of the countries, we use a similar approach as with the SHIHD. That is, we compute ratios of subnational HIV prevalence to national HIV prevalence of the year closest to the year for which we have the indicators available at both levels. We use these ratios to estimate subnational HIV prevalence from national HIV prevalence for the years when only national HIV prevalence data is available. For countries for which no subnational-level HIV data are available (i.e., Central African Republic, Comoros, Madagascar, Sudan and Swaziland), we use national-level estimates for all regions in those countries. We extrapolate the data for years between 1970 and 1990 according to an exponential growth model starting at zero in 1970, assuming that HIV spreads exponentially (Faria et al. 2014; Morris and Kretzschmar 1997). We set the HIV prevalence indicators to zero for all years before 1970, as HIV was not identified before this time.

### Missing Data

We use multiple imputation by chained equations (MICE) to deal with missing values. Figures S8 and S7 in SI H show aggregation plots for missingness in our aggregated subnational-level data. The variables with the highest proportion of missing values are the average ages at first marriage (18%) and sex (23%), polygyny (18%) and marital status (18%) for women. For men, age at first birth has the highest proportion of missingness (33%). Most of the missingness in our data is the result from particular questions not being asked in certain surveys (such as the age at first childbirth for men) or from missingness in external data (such as the HIV and SHDI/HIHD data). MICE replaces missing values in a data set under certain assumptions about the missingness mechanism, and imputes these values *M* times (creating *M* different data sets) to take into account the uncertainty of imputation (for more information, see Azur et al. 2011). The algorithm loops through the data, imputing every variable by means of predicting it using the other variables, and repeating this cycle as the data are updated. The statistical analyses are performed on each of these *M* data sets and then combined (i.e., pooled) as explained in SI J. The number of imputations used equals the percentage of incomplete observations (i.e., region-year combinations for which at least one variable is missing) in the female (M = 28) and male (M = 34) data, as recommended by Bodner (2008); White et al. (2011). As multiple imputation incorporates between-data uncertainty resulting from the imputation (i.e., estimation) of missing values in the standard errors, accurate and unbiased estimates will be produced.

## Methods

Our analysis proceeds in four steps. First, we document descriptive levels and trends of childlessness across sub-Saharan Africa. Second, we examine the bivariate relationship between childlessness and development on both the national and subnational levels. Third, we estimate two-level hierarchical models to examine whether childlessness and development relate according to a U-shape. Fourth, we split overall childlessness into its types (i.e., involuntary, voluntary and circumstantial) and overall development into its components (i.e., life expectancy, education and income). We then examine the bivariate relationships between these types and components to investigate the drivers and mechanisms of the association between overall childlessness and development.

In the third part of our analyses, we estimate two-level hierarchical (i.e., multilevel) models where subnational regions are nested within countries. We use hierarchical modeling to take the clustered structure of our data into account (for a more detailed explanation, see Snijders and Bosker 2011). Our model is specified as

4 $$\begin{aligned} Childlessness_{trc} = \pi _{0c} + {\underline{\pi }}_{1c} {\underline{SHIHD}}_{trc} + {\underline{\pi }}_{2}{\underline{X}}_{trc} + e_{trc} \end{aligned}$$

where *r* and *c* represent subnational regions and countries, respectively, *t* represents time (year minus sample minimum year) and underlined objects represent vectors. Our independent

5 $$\underline{{SHIHD}} _{{trc}} = \left[ {{\text{poly}}\left( {SHIHD_{{trc}} ,1} \right),{\text{poly}}\left( {SHIHD_{{trc}} ,2} \right)} \right]^{\prime }$$

has dimension $(2 \times 1)$ and represents the second-order polynomials of the SHIHD term. Note that we use orthogonal rather than raw polynomial terms of SHIHD in the regression. As the raw polynomial terms of SHIHD (i.e., $SHIHD_{trc}$, $SHIHD^2_{trc}$, $SHIHD^3_{trc}$, etc.) are highly correlated with each other, the resulting standard errors would be inflated due to multicollinearity, yielding larger confidence intervals and therefore biased significance tests for the regression coefficients. As a consequence, the regression results might lead us to incorrectly conclude that there is no U-shape in the relationship between SHIHD and childlessness, while in reality there is. To avoid this from happening, we use orthogonal (i.e., uncorrelated) rather than raw polynomial terms of SHIHD (see SI I or Kennedy and Gentle (p. 343-344, 1980) for a more detailed explanation of how these terms are computed).

Note that $Childlessness_{trc}$, $\pi _{0c}$ and $e_{trc}$ are scalars, whereas ${\underline{\pi }}_{2}$ and ${\underline{\pi }}_{1c}$ are vectors of length two and *k*, respectively. Further, ${\underline{X}}_{trc}$ is a matrix of dimension $(k \times 1)$ containing *k* explanatory variables for time *t*, region *r* in country *c*. In our baseline model (M1), ${\underline{X}}_{trc}$ only consists of the second-order orthogonal polynomials of the time variable ($k=2$), which we add to the model to capture secular time trends (i.e., changes in childlessness over time that are not captured by changes in the SHIHD). In models M2 through M4, we add sets of variables to the ${\underline{X}}_{trc}$ of M1, namely either average age and proportion of urban residence in M2 $(k=4)$; marital status and postponement in M3 $(k=8)$; polygyny and HIV prevalence as well as marriage market sex balance in M4 $(k=6)$. In M5, ${\underline{X}}_{trc}$ contains all variables included in ${\underline{X}}_{trc}$ throughout models M1 to M4. The correlation plots in Figures S9 and S10 in SI K show that multicollinearity should not pose any problems in the analyses.

We allow the intercept $\pi _{0c}$ and coefficients $\pi _{1c} = [{\pi _{11c}, \pi _{12c}}]$ for the second-order orthogonal SHIHD terms to vary across countries, as is standard procedure in multilevel modeling (Snijders and Bosker 2011). With this specification, the residual variance can be partitioned into between-country (country-level) and within-country (subnational-level) components. The country-level residuals stand for unobserved country characteristics that result in correlated outcomes for regions within the same country. More formally,

6 $$\begin{aligned} \begin{aligned} \pi _{0c}&= \gamma _{00} + \gamma _{01} W_{tc} + u_{0c}\\ \pi _{11c}&= \gamma _{101} + \gamma _{111} W_{tc} + u_{11c}\\ \pi _{12c}&= \gamma _{102} + \gamma _{112} W_{tc} + u_{12c} \end{aligned} \end{aligned}$$

Note that in this specification $W_{tc}$ contains country-level variables, and so in our case $W_{tc}$ is a scalar containing the number of women per 100 men for country *c* at time *t*.

Our strategy for pooling the results from the regressions on all imputed data is explained in SI J. Note that we assess model fit using the pooled Akaike Information Criterion (AIC) and Bayesian Information Criterion (BIC), following the pooling approach of Knowles and Frederick (2018) according to ${\overline{IC}} = \sum _{i=1}^M IC_i$. As recommended by Burnham et al. (2011), we conclude one model is better than another if the AIC difference between the models is larger than nine.

As population estimates are not available on the subnational level and our goal is not necessarily to draw conclusions about the average sub-Saharan African case, we do not age-adjust these childlessness levels nor weigh the regressions for country size. As the absolute difference between age-adjusted and raw childlessness levels on the national level is 0.04% on average and 0.8% at maximum, this decision should not affect our conclusions.

## Results

**Fig. 3:**
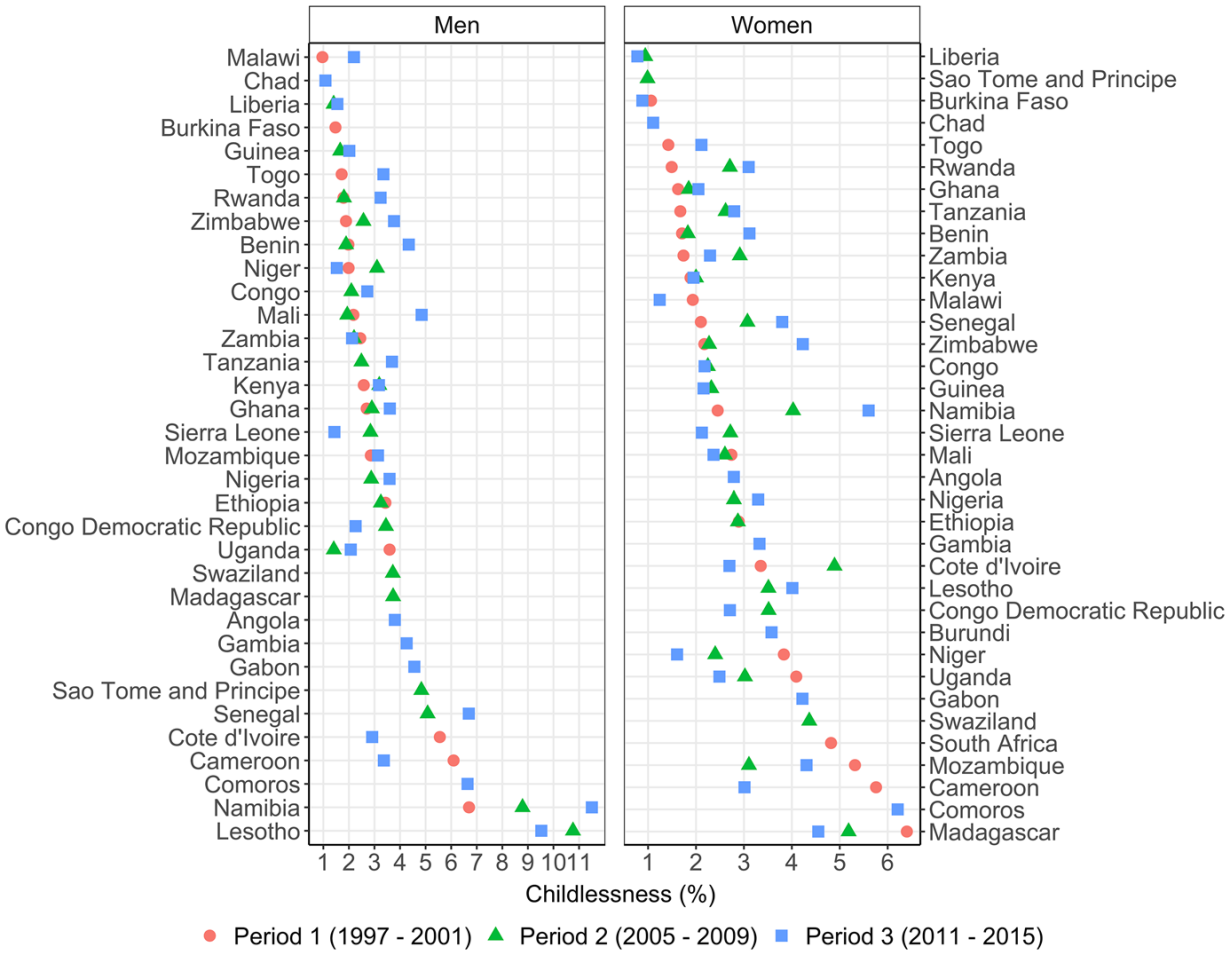
National childlessness levels for men and women in 1997-2001 (red circle), 2005–2009 (green triangle) and 2011–2015 (blue square)

We first document national-level childlessness trends by gender. Figure 3 shows the level of childlessness for three periods: 1997–2001 (red circle), 2005-2009 (green triangle) and 2011–2015 (blue square);^4^ the raw trends are shown in Figure S11 in SI L. We observe that national levels of childlessness vary between almost one percent and 12 percent. Some of these highest levels of childlessness occur among men rather than women, and there is also more variation across time and place in male than female childlessness. For each of the three periods considered, male childlessness is higher than female childlessness for about 60 to 65% of the countries for which both female and male surveys were collected. There is substantial variation across countries in the trends in childlessness. Over the periods considered, we observe increases in male childlessness in 14 countries, whereas 11 countries show a stable pattern or decrease. For female childlessness, we observe increasing trends in 11 countries, and stable or decreasing trends in 16 countries. Note that while the time between two surveys collected for a given country will usually be sufficient (see Figure S1 in SI B), the time differences between the periods might be small in some cases.

**Fig. 4:**
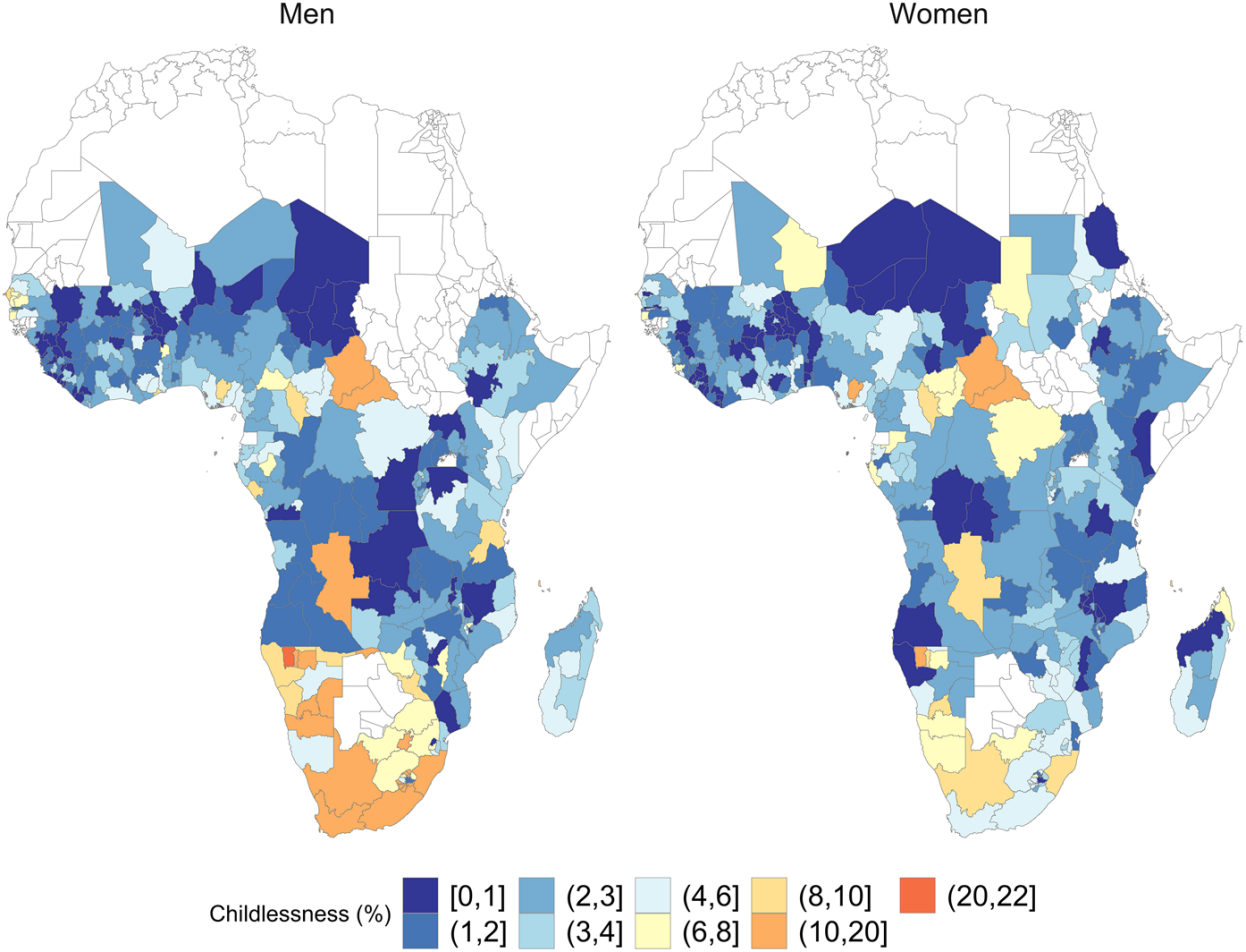
Male and female childlessness across 372 respectively 384 subnational regions in sub-Saharan Africa

Moving to the sub-national level, Fig. 4 shows the most recently available level of childlessness separately for men and women in 372 and 384 regions, respectively. The unweighted average for male childlessness is 3.5%, slightly higher than the average for females of 2.9%. Although there is substantial variation, particularly among men, at least one fifth of the subnational regions have childlessness levels exceeding 5%. Regions with some of the highest female childlessness levels ($\sim$16%) include Addis in Ethiopia as well as Abia, Borno, Imo and Anambra in Nigeria. These regions have in common that they either include the country capital, or that they are some of the most populated areas in their country. However, some of the highest levels of childlessness do not occur among women, but rather among men. In some regions in South Africa (Western Cape), Central African Republic (Basse Kotto, Mbornou, Houte Mbormo), and Namibia (Omusati, Caprivi, Khomas and Oshana), male childlessness levels achieve between 16 and 33%. These are regions which are densely populated compared to other regions in the same country, or regions where infertility has generally been high (Larsen 2003). In 215 (56%) of the regions for which both male and female data are available, the most current levels of male childlessness are higher than those for female childlessness. In 30 out of those 215 regions (14%), childlessness among men is at least 5 percentage points (p.p.) higher than among women.

**Fig. 5:**
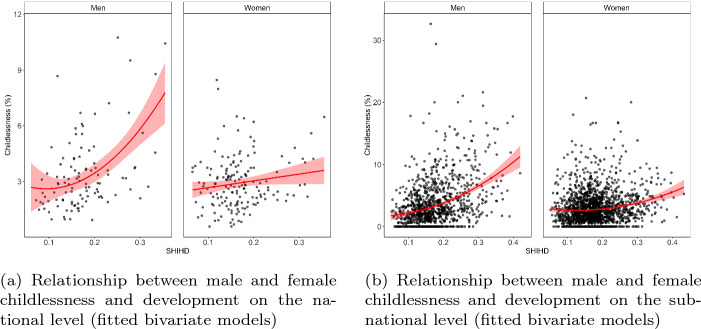
Relationship between male and female childlessness and development on the **a** national and **b** subnational level (fitted bivariate models)

What does the relationship between overall levels of childlessness and development look like, and (how) is this different on the national and subnational levels? In Figs. 5a and 5b, we visualize the relationship between childlessness and development on the national, respectively, subnational levels by means of scatter plots and fitted lines representing the best performing of linear or quadratic bivariate models. Note that in all scatter plots, the points represent combinations of countries or subnational regions with years and are slightly transparent to better distinguish between overlapping points. We observe from Figs. 5a and 5b that on the subnational level, we have much more variation in both childlessness levels and development than on the national level. On the national level, the bivariate association between childlessness and development is best described as a quadratic U-shaped relationship for men and a linear relationship for women. On the subnational level, where we have much more variation, both female and male childlessness associate with development according to a U-shape. These observations are supported by the model fits in Tables S2 and S3 in SI M.

As we have a much larger sample of subnational region-year combinations ($N_{men} =$ 1,213 and $N_{women} =$ 1,749) than we do of national country-year combinations ($N_{men} =$ 119 and $N_{women} =$ 165) and there is substantial within-country variation in both childlessness and development, we continue our analysis from a subnational perspective. In doing so, we estimate five hierarchical models for women and men separately. We allow the intercept and effect of the first-order orthogonal polynomial term of SHIHD to vary across countries because it significantly improves the model fit (see Table S5 in SI N). Further, the results for the controlled models with random effects showed that linear models fit better for men, while quadratic models fit better for women (Table S4 in SI M). Therefore, our multivariate models include only first-order orthogonal polynomials (1$^{st}$ OP) of SHIHD for men but also second-order orthogonal polynomials of SHIHD (2$^{nd}$ OP) for women. Table S6 in SI O contains the random effects. These show that the effect(s) of the SHIHD polynomial(s) vary substantially across countries—supporting our choice for random effects models—and that there is a positive correlation between intercept and the effect of the SHIHD polynomial(s), indicating that countries with higher levels of childlessness at baseline also experience a steeper growth in childlessness as development increases.

**Fig. 6:**
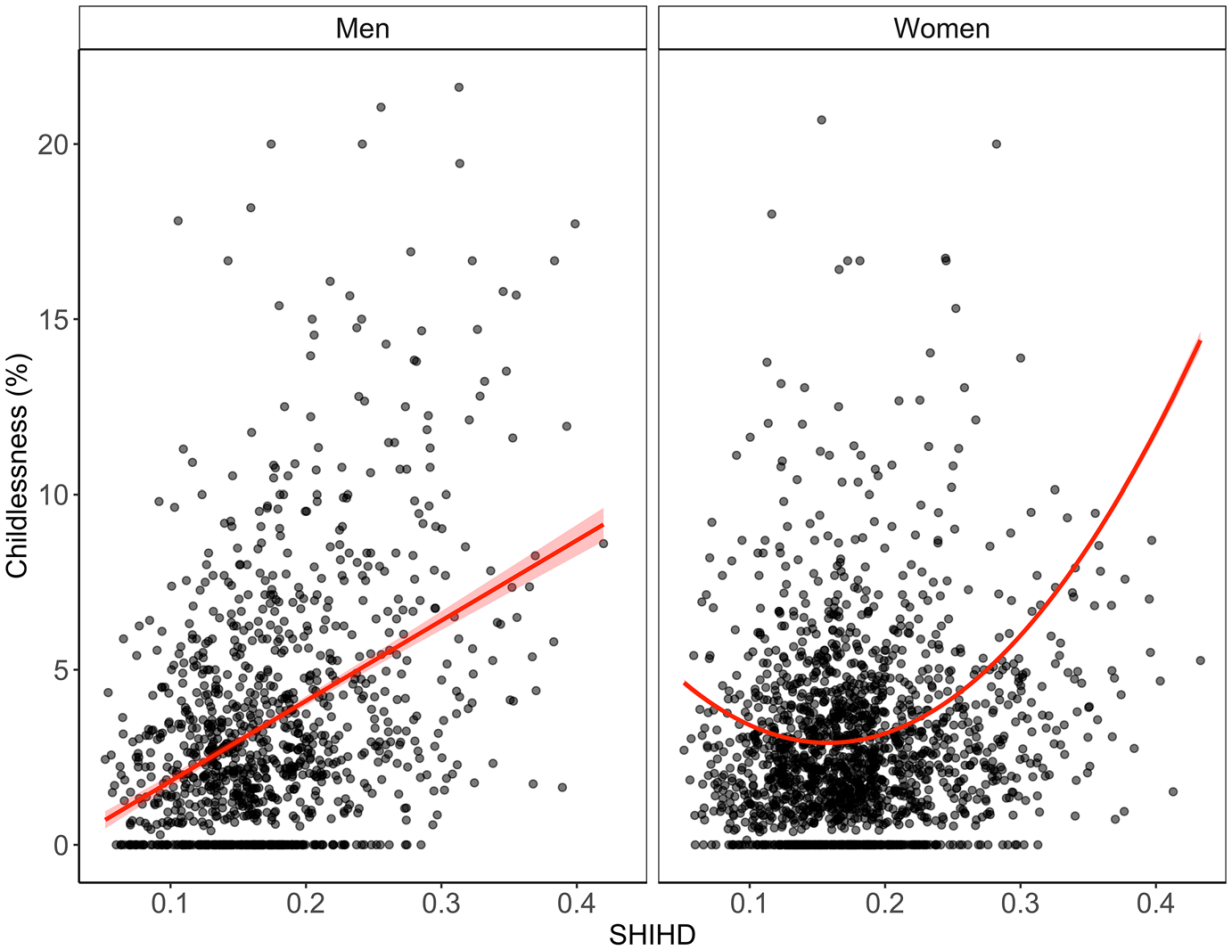
Fitted values for the multivariate regressions estimating male and female childlessness from development on the subnational level (model M5)

Does the relationship between childlessness and development remain when taking other factors into account? Fig. 6 shows the predicted relationship between childlessness and development for the full control model (M5). We observe that the relationship between female childlessness and development is indeed U-shaped. The hierarchical model results in Table 3 further show that development is still related to childlessness even when controlling for other factors. For men, this is not the case, however. Instead of a U-shaped pattern, we observe that a positive linear function describes the relationship between male childlessness and development (Fig. 6). Additionally, in contrast with the results for women, overall levels of development are not statistically significantly related to male childlessness when controlling for other factors (Table 3). Further, there is no secular time trend for men, whereas the first- and second-order orthogonal polynomials of time are generally statistically significant throughout the models for women.

While overall levels of development are not statistically significantly associated with male childlessness, do any of the other factors in our models show significant correlations? Table 3 shows the regression coefficients for the subnational-level multivariate hierarchical models estimating childlessness. Figure S12 in SI P shows the marginal effect of each of the independent variables (ceteris paribus) for the full control model (M5, linear for men and quadratic for women) to give a sense of the (relative) size and nature of the model associations, given that the regression coefficients may be difficult to interpret directly because of the hierarchical nature of the models and the use of orthogonal polynomials. Among men, childlessness seems to be driven mainly by postponement or even a complete lack of marriage, given that the effect of SHIHD is not statistically significant in the models controlling for marital status and postponement indicators. Male childlessness is substantially larger in regions where singlehood and divorce are more common relative to marriage. However, it is not only the lack of marriage but also the postponement of marriage by men that leads to higher levels of male childlessness, given the statistically significant positive coefficients for the average age at first marriage or cohabitation for males. Taking into account the range of values occurring in the data for each of the independent variables, Figure S12a in SI P shows that the prevalence of male singlehood has by far the strongest relationship with male childlessness: for example, an increase from 20% to 40% of never married men is associated with an average increase from 12% to 22% in male childlessness. Similarly, female childlessness is also driven by divorce, although among women the prevalence of singlehood does not affect childlessness when controlling for other factors. Rather, higher prevalences of HIV as well as urbanization are associated with female childlessness. Another factor strongly related to both male and female childlessness is polygyny: childlessness is substantially lower in regions where polygyny is more common. For women, however, SHIHD is most strongly related to childlessness compared to other predictors, considering the realistically occurring values of these predictors (Figure S12b in SI P). To illustrate, while there is substantial variation across subnational regions, on average the SHIHD across these regions increased from approximately 0.1 around 1990 to 0.2 around 2010. This increase in development would be associated with an initial decrease from 4% to 2% followed by an increase to 3%. For some of the more developed regions, which have gone from about 0.2 to 0.3 or 0.3 to 0.4 on the SHIHD scale in the same period, female childlessness would have increased on average from about 2-3% to 5-6%, respectively, 5-6% to 11-12%.

**Table 3:** Regression coefficients of the subnational regional-level multivariate hierarchical models estimating childlessness from development for men and women

	Men (*N* = 1,239)					Women (*N* = 1,775)				
	Model 1	Model 2	Model 3	Model 4	Model 5	Model 1	Model 2	Model 3	Model 4	Model 5
Intercept	0.035$$^{***}$$	$$-$$0.025	$$-$$0.030$$^{*}$$	0.017	$$-$$0.071	0.036$$^{***}$$	0.036	0.030$$^{*}$$	0.118$$^{**}$$	0.094
	(0)	(0.464)	(0.045)	(0.766)	(0.163)	(0)	(0.280)	(0.021)	(0.007)	(0.111)
Time (1st OP)	$$-$$0.019	0.018	$$-$$0.030	$$-$$0.022	$$-$$0.003	$$-$$0.078$$^{**}$$	$$-$$0.061$$^{*}$$	$$-$$0.070$$^{*}$$	$$-$$0.079$$^{*}$$	$$-$$0.082$$^{*}$$
	(0.675)	(0.674)	(0.457)	(0.646)	(0.941)	(0.009)	(0.040)	(0.033)	(0.021)	(0.030)
Time (2nd OP)						0.069$$^{**}$$	0.063$$^{*}$$	0.067$$^{*}$$	0.076$$^{**}$$	0.057$$^{*}$$
						(0.007)	(0.013)	(0.012)	(0.003)	(0.034)
SHIHD (1st OP)	0.404$$^{***}$$	0.380$$^{***}$$	0.054	0.293$$^{**}$$	0.005	0.434$$^{**}$$	0.331$$^{*}$$	0.339$$^{*}$$	0.359$$^{*}$$	0.224
	(0)	(0)	(0.449)	(0.004)	(0.945)	(0.008)	(0.040)	(0.038)	(0.026)	(0.155)
SHIHD (2nd OP)						0.396$$^{***}$$	0.368$$^{***}$$	0.396$$^{***}$$	0.395$$^{***}$$	0.359$$^{***}$$
						(0.001)	(0.001)	(0)	(0)	(0.001)
Average age		0.001$^{+}$			0.001		0			0
		(0.091)			(0.176)		(0.918)			(0.916)
Urban residence (%)		0.003			0.003		0.008$$^{**}$$			0.006$$^{*}$$
		(0.456)			(0.464)		(0.006)			(0.036)
Never married^a^ (%)			0.498$$^{***}$$		0.493$$^{***}$$			0.015$$^{+}$$		0.011
			(0)		(0)			(0.068)		(0.205)
Divorced/separated^a^ (%)			0.072$$^{***}$$		0.069$$^{***}$$			0.049$$^{***}$$		0.035$$^{*}$$
			(0.001)		(0.001)			(0.001)		(0.018)
Widowed^a^ (%)			0.073$^{+}$		0.060			$-$0.008		$-$0.014
			(0.076)		(0.142)			(0.559)		(0.292)
Average age at first birth			0		0			0		0
			(0.356)		(0.393)			(0.897)		(0.268)
Average age at first			0.001$^{*}$		0.001$^{*}$			0.001$^{+}$		0.001
marriage/cohabitation			(0.017)		(0.035)			(0.051)		(0.156)
Average age at first sex			0		0			$-$0.001		$-$0.001
			(0.967)		(0.957)			(0.133)		(0.139)
Polygyny prevalence (%)				$$-$$0.035$$^{***}$$	$$-$$0.014$$^{+}$$				$$-$$0.015$$^{**}$$	$$-$$0.013$$^{*}$$
				(0)	(0.074)				(0.001)	(0.015)
HIV prevalence (%)				0.020	0				0.040$^{+}$	0.040$^{+}$
				(0.860)	(0.998)				(0.095)	(0.097)
Number of women				0	0				$-$0.001$^{+}$	$-$0.001
per 100 men				(0.664)	(0.910)				(0.073)	(0.205)
AIC	$-$4927.427	$-$4907.640	$-$5191.312	$-$4914.396	$-$5141.966	$-$8097.594	$-$8081.931	$-$8060.544	$-$8089.681	$-$8018.600
BIC	$-$4891.721	$-$4861.732	$-$5125.001	$-$4863.388	$-$5050.151	$-$8031.992	$-$8005.396	$-$7962.141	$-$8007.679	$-$7892.864
Log. Lik.	2470.713	2462.820	2608.656	2467.198	2588.983	4060.797	4054.965	4048.272	4059.841	4032.300

^a^ The reference category for marital status is “married, cohabiting or in a relationship.”

$$^{+}p<0.1; ^{*}p<0.05; ^{**}p<0.01; ^{***}p<0.001$$

**Fig. 7:**
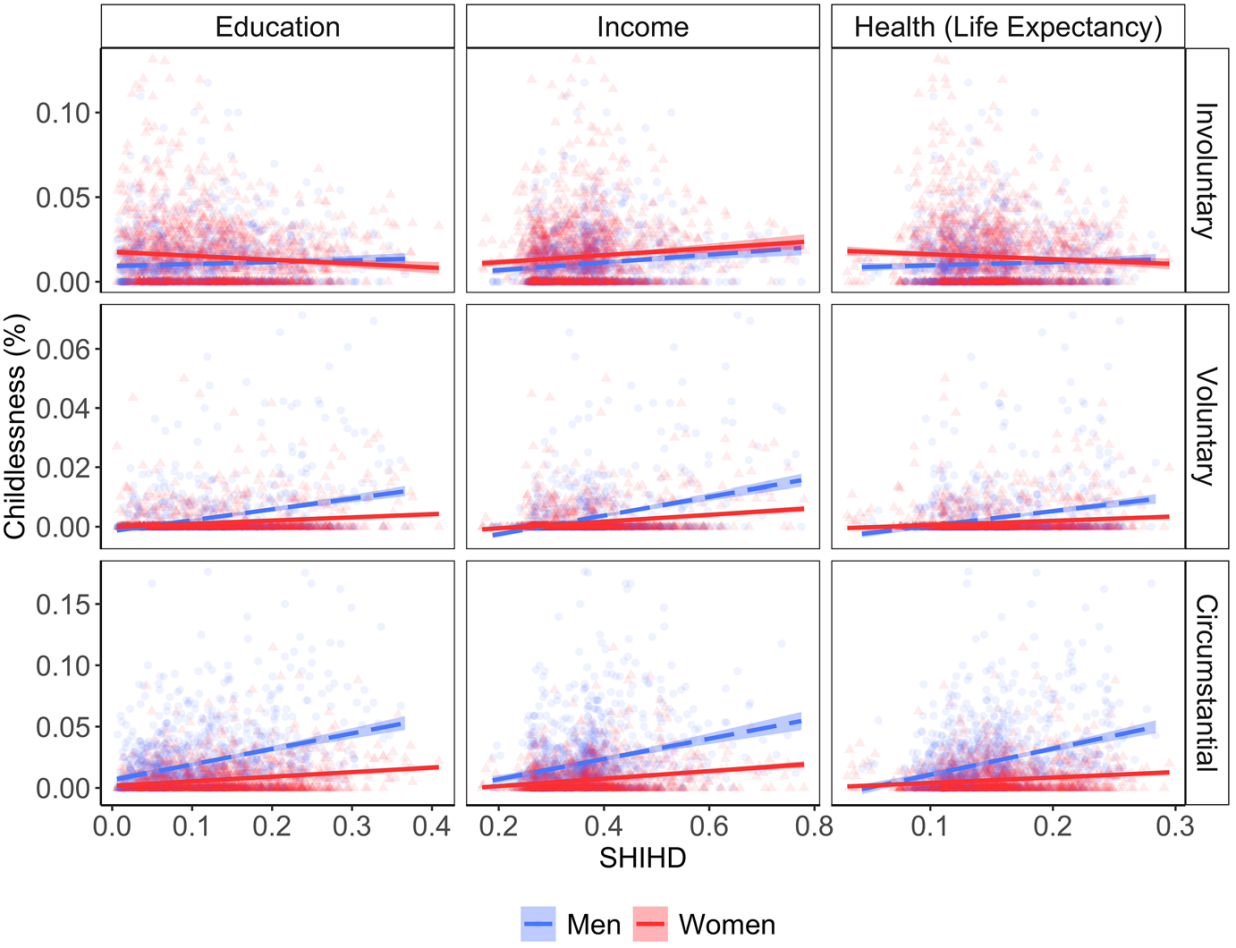
Fitted values for the regressions estimating male and female childlessness types from development components on the subnational level

What drives the U-shape between female childlessness and development? And why do we not observe such a U-shape among men? To answer these questions, we estimate linear bivariate models between childlessness types (i.e., involuntary, voluntary and circumstantial) and development components (i.e., life expectancy, education and income) according to our hypotheses in Fig. 1. Figure 7 shows the plotted fitted values and Table S7 in SI Q contains the regression coefficients. We estimate linear models because we have no reason to believe that childlessness types and development components are nonlinearly related, and we expect that these linear relationships together result in an overall U-shape in the association between childlessness and development (as illustrated in Fig. 1). We observe that voluntary and circumstantial childlessness are positively related with life expectancy and particularly education and income, especially among men. Further, involuntary childlessness and income are positively associated for both men and women. The associations of involuntary childlessness with education and life expectancy are different for men and women, however: while female involuntary childlessness decreases with education and life expectancy, male involuntary childlessness remains unchanged with life expectancy and even increases slightly with education.

## Discussion and Conclusion

Although childlessness has gained increasing interest from researchers and policy makers, little was known about childlessness in sub-Saharan Africa. We filled this gap by presenting the first comprehensive overview of male and female childlessness in relation with development in sub-Saharan Africa. Moreover, we constructed a subnational historical index of development to examine the relationship between childlessness and development at the subnational level. We were able to exploit maximum variation in development and take into account substantive within-country variation in development and childlessness. We also analyzed the relationship between different types of childlessness and development components as well as the gender differences in this relationship.

We have firstly shown that permanent childlessness in sub-Saharan Africa is substantial, particularly among men. This finding contradicts the common perception that childlessness in sub-Saharan Africa is mainly a female “issue” and that it hardly occurs among men (World Health Organization 2014). This evidence stresses the importance not only of further expanding research on childlessness in sub-Saharan Africa, but also focusing on males besides females in doing so. Additionally, we have been able to distinguish between different types of childlessness to shed more light on the prevalence of childlessness not stemming from infertility. We have shown that although involuntary childlessness is still the most common type of childlessness in sub-Saharan Africa (Baudin et al. 2020), particularly among women, voluntary and especially circumstantial childlessness also prevail, and more so with increasing levels of development. By distinguishing between different types of childlessness and different components of development, we were also able to further investigate the drivers behind the observed U-shaped relationship between overall levels of female childlessness and development. We found that involuntary childlessness is negatively associated with life expectancy and education, although only among women, and that voluntary and circumstantial childlessness positively relate with life expectancy and particularly education and income among both men and women. These findings are in line with previous studies for other low-income regions (Poston et al. 1985; Poston and Trent 1982; Poston and El-Badry 1987; Baudin et al. 2020) and seem to suggest that the observed U-shape indeed is the result of involuntary childlessness declining with life expectancy and education, and voluntary and circumstantial childlessness associating positively with life expectancy and particularly education and income. On the one hand, these findings highlight the importance of taking additional measures to further reduce poverty and infertility. On the other hand, increasing shares of individuals not partnering or choosing not to have children might support speculations that the drivers of childlessness in high-income countries are operating, although perhaps to a smaller extent, in sub-Saharan Africa too (Wilson 2011).

For men, however, we do not find any evidence for a U-shaped relationship, but rather a positive linear association. This may perhaps be the result of the absence of negative associations of male involuntary childlessness with life expectancy and education, which we do observe for women. Why might we not observe these negative associations for men? Under-reporting infertility may be more common among men as infertile men even more than women carry the burden of being stigmatized for a lack of virility and sexual impotency (Agarwal et al. 2015; Runganga et al. 2001). Additionally, male infertility may actually have increased with development as a consequence of rises in chemical exposure, stress, psychoses, drug and alcohol abuse, epidemics, hormone imbalance and tobacco consumption, a phenomenon we have also observed in more developed regions (Poston and Kramer 1983; Sengupta et al. 2017). Interestingly, the positive relationships of voluntary and circumstantial childlessness with life expectancy, education and income are stronger among men than women. This finding corresponds to the expectation that more low-status men will be left behind on the marriage market as women emancipate (van Bavel 2012; Chudnovskaya 2019; Kreyenfeld and Konietzka 2017), but contradicts our hypotheses that advancements in education and income have a greater impact on women and advancements in life expectancy affect men and women to a similar extent. A possible explanation for this finding may be perhaps that sub-Saharan Africa still knows relatively low levels of gender equality, and that the positive effects of education and income are still more pronounced among men than women (Browne and Millington 2015).

While for female childlessness the positive relationship with overall levels of development remains even when controlling for other factors, male childlessness can rather be explained, in a statistical sense, by changes in marital status and polygyny. Our multivariate models suggest that male childlessness in sub-Saharan Africa is mainly driven by a lack of marriage, which seems plausible because childbearing in sub-Saharan Africa is usually higher among married couples than among single or divorced individuals (Rutstein and Shah 2004). Another important driver for childlessness is polygyny. Higher polygyny prevalence is associated with lower male childlessness, which makes sense simply because men in polygamous unions have more female partners and thus chances to have children with (Rossi 2019; Schoumaker 2019). Surprisingly, we find that higher polygyny also relates to lower female childlessness, while we had expected polygyny to positively associate with female childlessness through lower frequency of sexual intercourse, higher chances of venereal diseases and the selection of infertile women into polygynous unions (Cahu et al. 2014; Lardoux and van de Walle 2003; Musham 1956). Perhaps in regions where polygyny is common, childbearing is much more important and desired and daughters are seen as a good investment, thereby increasing the willingness to have a child as well as the competition between co-wives to bear children (Rossi 2019; Tertilt 2005).

Our study has some limitations that need to be considered. First, a common difficulty is to assess permanent childlessness among men, as the end of the reproductive period is not as clearly marked for men as it is for women. To retain sufficient sample sizes, we used a cutoff age of 40. Although our results are robust to using higher cutoffs up to the mid-40s, we cannot fully rule out that this early cutoff age produces a small upward bias in our estimates, especially for men. Second, and perhaps more importantly, our estimates of childlessness may be downwardly biased as a consequence of over-reporting fertility due to the stigma attached to childlessness in sub-Saharan Africa (van Balen and Bos 2009; Larsen 1989). Similarly, voluntary childlessness may be overestimated as a consequence of ex-post rationalization, whereby responses to questions about fertility ideals are aligned to one’s actual fertility outcomes to mitigate social and individual hardship that may stem from childlessness (Pritchett 1994). Although men may equally over-report fertility, they may also under-report fertility if they are not aware of having produced children (Rendall et al. 1999; Schoumaker 2017). Under-reporting may also occur when parents do not include the birth of a child who has died. Note, however, that the DHS explicitly ask for all live births regardless of survival. Moreover, while this may affect the total number of children, it is unlikely to affect childlessness status. To limit bias from misreporting fertility, we use the “total number of (own) children ever born” variable to estimate childlessness, which has been shown to be the most accurate variable to be used for estimating fertility in the DHS data (Schoumaker 2017). Finally, we may have overestimated childlessness because we cannot include women who deceased during pregnancy or delivery. We were not able to account for such selection because accurate measures of maternal mortality were not sufficiently available. We refrain from speculating about the sum of these limitations; they may cancel each other out or produce a small bias.

What are the implications of our results for how we might expect childlessness rates to evolve with future levels of development in sub-Saharan Africa? It seems plausible that involuntary childlessness will continue to decrease with further progress in life expectancy and education. One of the most interesting questions is whether circumstantial and voluntary childlessness will follow patterns observed in high-income countries, especially among higher educated women in urbanized areas. On the one hand, we might expect similar patterns and some level of convergence as the main drivers of childlessness in high-income countries – female education and labor market position, technological change, increasing wealth and autonomy, and urbanization – seem to be operating in sub-Saharan Africa too (Wilson 2011). On the other hand, despite similarity in these large socioeconomic forces, it has been both argued and documented that patterns observed in more developed countries need not simply apply in a straightforward way to low- and middle-developed countries as they become more developed (e.g., Esteve and Florez-Paredes 2018; Furstenberg Jr 2013; Thornton 2013). Moreover, although our analyses highlight the important role marriage systems play in childlessness, heterogeneity in family change within and between African countries (Pesando and GFC team 2018) suggests we should not expect a pattern of universal convergence of childlessness types across the region. Our study has shown that (changes in) childlessness in sub-Saharan Africa are associated with development, but that there is much variation herein. Both observations call for further analyses with a focus on heterogeneity in developments within sub-Saharan Africa. Empirical and conceptual challenges have to be addressed to make progress on this topic. Conceptually, we need a framework that acknowledges that structural change in development is embedded in different contexts and therefore may result in different outcomes. Empirically, for instance, we may need to turn to other data sources that provide enough statistical power to explore sub-populations and geographic variation in sufficient detail. Still, for our current level of analysis, new rounds of DHS will show us whether the U-shape becomes more pronounced or not with increasing levels of development.

## Supplementary Information

Below is the link to the electronic supplementary material.

Supplementary file 1 (pdf 11764 KB)

## Data Availability

The code used to generate the results presented in this paper is available on Github (https://github.com/fverkroost/ejp-childlessness-development-ssa). The datasets analyzed during the current study are available in the DHS Program repository, (https://dhsprogram.com/data/available-datasets.cfm). The indicators of development are constructed from the Historical Index of Human Development (Prados de la Escosura 2015a), (https://espacioinvestiga.org/home-hihd/hihd-downloads/?lang=en) and the Subnational Human Development Index (Smits and Permanyer 2019). (https://globaldatalab.org/shdi/shdi/) Marriage market sex balances are computed using the United Nations sex-specific 2017 World Population Prospects (United Nations Desa/Population Division 2017a, b). HIV prevalence data are obtained from the Joint United Nations Programme on HIV/AIDS (UNAIDS) (2017a) (in the case of national-level data) and from the US Agency for International Development (2018) and Joint United Nations Programme on HIV/AIDS (UNAIDS) (2017b) (in the case of subnational-level data).

## Footnotes

1 In this paper, we define infertility as “the inability to bear any children, either due to the inability to conceive or the inability to carry a pregnancy to a live birth” (Rutstein and Shah 2004, p. 3).

2 The age distributions for women and men per country are shown in Figure S3 in SI D.

3 Czech Republic and Luxembourg were excluded from these calculations because data on the HIHD and its components were missing for these countries.

4 The first, second and third quartiles of the year of observation across surveys are 1999, 2007 and 2013, respectively. As some surveys are collected not exactly in but close to these years, we consider surveys collected as closely as possible to each of these quartiles within two years before or after one of the quartiles, resulting in the ranges 1997–2001, 2005–2009 and 2011–2015.

## References

[CR1] Agarwal A, Mulgund A, Hamada A, Chyatte MR (2015). A unique view on male infertility around the globe. Reproductive Biology and Endocrinology.

[CR2] Azur MJ, Stuart EA, Frangakis C, Leaf PJ (2011). Multiple imputation by chained equations: What is it and how does it work?. International Journal of Methods in Psychiatric Research.

[CR3] van Balen F (2008). Involuntary childlessness: A neglected problem in poor-resource areas. ESHRE Monographs.

[CR4] van Balen F, Bos HM (2009). The social and cultural consequences of being childless in poor-resource areas. Facts, Views and Vision in ObGyn.

[CR5] Barnard J, Rubin DB (1999). Small-sample degrees of freedom with multiple imputation. Biometrika.

[CR6] Baudin T, De La Croix D, Gobbi PE (2015). Fertility and childlessness in the United States. American Economic Review.

[CR7] Baudin T, de la Croix D, Gobbi PE (2020). Endogenous childlessness and stages of development. Journal of the European Economic Association.

[CR89] van Bavel J (2012). The reversal of gender inequality in education, union formation, and fertility in Europe. Vienna Yearbook of Population Research.

[CR90] van Bavel J, Kok J (2010). Pioneers of the modern lifestyle?: Childless couples in the early-twentieth-century Netherlands. Social Science History.

[CR8] Beaujouan E, Brzozowska Z, Zeman K (2016). The limited effect of increasing educational attainment on childlessness trends in twentieth-century Europe, women born 1916–65. Population Studies.

[CR9] Becker G (1960). An economic analysis of fertility. Demographic and economic change in developed countries.

[CR10] Becker G (1981). A treatise on the family.

[CR11] Bodner TE (2008). What improves with increased missing data imputations?. Structural Equation Modeling.

[CR12] Bongaarts J, Casterline J (2013). Fertility transition: Is sub-Saharan Africa different?. Population and Development Review.

[CR13] Browne, E., & Millington, K. A. (2015). Social development and human development: Topic guide. Governance and Social Development Resource Centre (GSDRC). Retrieved from: https://gsdrc.org/wp-content/uploads/2015/10/SD_HD.pdf.

[CR14] Burkimsher M (2014). Is religious attendance bottoming out? An examination of current trends across Europe. Journal for the Scientific Study of Religion.

[CR15] Burkimsher M, Zeman K, Kreyenfeld M, Konietzka D (2017). Childlessness in Switzerland and Austria. Childlessness in Europe: Contexts, causes, and consequences.

[CR16] Burnham KP, Anderson DR, Huyvaert KP (2011). AIC model selection and multimodel inference in behavioral ecology: Some background, observations, and comparisons. Behavioral Ecology and Sociobiology.

[CR17] Cahu, P., Fall, F., & Pongou, R. (2014). Beauty, polygyny and fertility: Theory and evidence. MPRA Paper No. 59009. Retrieved from: http://mpra.ub.uni-muenchen.de/59009/.

[CR18] Caselli G, Vallin J, Wunsch G (2005). Demography: Analysis and Synthesis: A Treatise in Population.

[CR19] Casterline J, Han S (2017). Unrealized fertility: Fertility desires at the end of the reproductive career. Demographic Research.

[CR20] Chudnovskaya M (2019). Trends in childlessness among highly educated men in Sweden. European Journal of Population.

[CR21] Clark S, Brauner-Otto S (2015). Divorce in sub-Saharan Africa: Are unions becoming less stable?. Population and Development Review.

[CR22] Dunlop CL, Benova L, Campbell O (2018). Effect of maternal age on facility-based delivery: Analysis of first-order births in 34 countries of sub-Saharan Africa using demographic and health survey data. BMJ open.

[CR23] Dyer SJ, Abrahams N, Mokoena N, van der Spuy ZM (2004). You are a man because you have children: Experiences, reproductive health knowledge and treatment-seeking behaviour among men suffering from couple infertility in South Africa. Human Reproduction.

[CR26] Esteve, A., & Florez-Paredes, E. (2018). The stability paradox: Why expansion of women’s education has not delayed early union formation or childbearing in Latin America. *Studies in Family Planning,**49*(2), 127–142. 10.1111/sifp.12055 29749632

[CR27] Faria N, Rambaut A, Suchard M, Baele G, Bedford T, Ward M, Tatem A, Sousa J, Arinaminpathy N, Pépin J, Posada D, Peeters M, Pybus O, Lemey P (2014). The early spread and epidemic ignition of HIV-1 in human populations. Science.

[CR28] Fenske J (2015). African polygamy: Past and present. Journal of Development Economics.

[CR29] Frejka T, Kreyenfeld M, Konietzka D (2017). Childlessness in the United States. Childlessness in Europe: Contexts, causes, and consequences.

[CR30] Furstenberg FF (2013). Transitions to adulthood: What we can learn from the West. The Annals of the American Academy of Political and Social Science.

[CR31] Hegdahl HK, Fylkesnes KM, Sandøy IF (2016). Sex differences in HIV prevalence persist over time: Evidence from 18 countries in sub-Saharan Africa. PloS One.

[CR32] ICF International (1986–2018). Demographic and health surveys (various, 1986-2018) [Data sets]. Calverton, Maryland: ICF International [Distributor], 2019. Retrieved from: https://dhsprogram.com/data/available-datasets.cfm.

[CR33] Inhorn MC, Patrizio P (2015). Infertility around the globe: New thinking on gender, reproductive technologies and global movements in the 21st century. Human Reproduction Update.

[CR34] Joint United Nations Programme on HIV/AIDS (UNAIDS) (2017a). National HIV prevalence among young men and women, 15–24 [Data set]. Retrieved from: http://aidsinfo.unaids.org.

[CR35] Joint United Nations Programme on HIV/AIDS (UNAIDS) (2017b). Regional HIV prevalence among young men and women, 15–24 [Data set]. Retrieved from: http://aidsinfo.unaids.org.

[CR36] van de Kaa DJ (1996). Anchored narratives: The story and findings of half a century of research into the determinants of fertility. Population Studies.

[CR37] Kennedy WJ, Gentle JE (1980). Statistical computing.

[CR38] Knowles, J., & Frederick, C. (2018). merTools: Tools for analyzing mixed effect regression models. Retrieved from: https://CRAN.R-project.org/package=merTools, R package version 0.4.1.

[CR39] Kreyenfeld M, Konietzka D, Kreyenfeld M, Konietzka D (2017). Childlessness in East and West Germany: Long-term trends and social disparities. Childlessness in Europe: Contexts, causes, and consequences.

[CR40] Lardoux S, van de Walle E (2003). Polygyny and fertility in rural Senegal. Population.

[CR41] Larsen U, Lesthaeghe R (1989). A comparative study of the levels and differentials of sterility in Cameroon, Kenya, and Sudan. Reproduction and social organization in sub-Saharan Africa.

[CR42] Larsen U (1994). Sterility in sub-Saharan Africa. Population Studies.

[CR43] Larsen U (2000). Primary and secondary infertility in Sub-Saharan Africa. International Journal of Epidemiology.

[CR44] Larsen U (2003). Infertility in Central Africa. Tropical Medicine & International Health.

[CR45] van Leeuwen E, Prins JM, Jurriaans S, Boer K, Reiss P, Repping S, van der Veen F (2006). Reproduction and fertility in human immunodeficiency virus type-1 infection. Human Reproduction Update.

[CR46] Letherby G (2002). Childless and bereft: Stereotypes and realities in relation to voluntary and involuntary childlessness and womanhood. Sociological Inquiry.

[CR47] Madsen, J. B., Moslehi, S., & Wang, C. (2017). What has driven the great fertility decline in developing countries since 1960? *The Journal of Development Studies, 54*(4), 738–757.

[CR48] Marmot M (2005). Social determinants of health inequalities. The Lancet.

[CR49] Marston M, Slaymaker E, Cremin I, Floyd S, McGrath N, Kasamba I, Lutalo T, Nyirenda M, Ndyanabo A, Mupambireyi Z (2009). Trends in marriage and time spent single in sub-Saharan Africa: A comparative analysis of six population-based cohort studies and nine demographic and health surveys. Sexually Transmitted Infections.

[CR50] Mascarenhas MN, Flaxman SR, Boerma T, Stevens GA, van der Poel S (2012). National, regional, and global trends in infertility prevalence since 1990: A systematic analysis of 277 health surveys. PLoS Medicine.

[CR51] Morris M, Kretzschmar M (1997). Concurrent partnerships and the spread of HIV. Aids.

[CR52] Moultrie, T. A., Sayi, T. S., & Timæus, I. M. (2012). Birth intervals, postponement, and fertility decline in Africa: A new type of transition? *Population Studies,**66*(3), 241–258. 10.1080/00324728.2012.701660 22891624

[CR53] Musham, H. (1956). Fertility of polygamous marriages. *Population Studies,**10*(1), 3–16.

[CR54] Neyer G, Hoem JM, Andersson G, Kreyenfeld M, Konietzka D (2017). Education and childlessness: The influence of educational field and educational level on childlessness among Swedish and Austrian women. Childlessness in Europe: Contexts, causes, and consequences.

[CR55] Odimegwu C, Ndagurwa P, Mwiza GS, Ololade B (2018). Cohabitation in sub-Saharan Africa: A regional analysis. Southern African Journal of Demography.

[CR56] Oppenheimer, V. K. (1994). Women’s rising employment and the future of the family in industrial societies. *Population and Development Review, 20*(2), 293–342*.*

[CR57] Permanyer I, Smits J (2020). Inequality in human development across the globe. Population and Development Review.

[CR58] Permanyer I, Esteve-Palos A, Garcia J, McCaa R (2015). Human development index-like small area estimates for Africa computed from IPUMS-international integrated census microdata. Journal of Human Development and Capabilities.

[CR59] Pesando L, GFC team (2018). Global family change: Persistent diversity with development. Population and Development Review.

[CR60] Poston DL, El-Badry SM (1987). Modernization and childlessness among the governorates of the Arab Republic of Egypt. Journal of Biosocial Science.

[CR61] Poston DL, Kramer KB (1983). Voluntary and involuntary childlessness in the United States, 1955–1973. Social Biology.

[CR62] Poston DL, Trent K (1982). International variability in childlessness: A descriptive and analytical study. Journal of Family Issues.

[CR63] Poston DL, Kramer KB, Trent K, Yu MY (1983). Estimating voluntary and involuntary childlessness in the developing countries. Journal of Biosocial Science.

[CR64] Poston DL, Briody E, Trent K, Browning HL (1985). Modernization and childlessness in the states of Mexico. Economic Development and Cultural Change.

[CR24] Prados de la Escosura, L. (2015a). Historical index of human development. Retrieved from: https://espacioinvestiga.org/00REPO/TablasExcel_HIHD_Dimensiones/_Ing/HIHD.xls.

[CR25] Prados de la Escosura L (2015). World human development: 1870–2007. Review of Income and Wealth.

[CR65] Pritchett LH (1994). Desired fertility and the impact of population policies. Population and Development Review.

[CR66] Rendall, M. S., Clarke, L., Peters, H. E., Ranjit, N., & Verropoulou, G. (1999). Incomplete reporting of men’s fertility in the United States and Britain: A research note. *Demography,**36*(1), 135–144. 10036598

[CR67] Rindfuss RR, Bumpass LL (1976). How old is too old? Age and the sociology of fertility. Family Planning Perspectives.

[CR68] Rossi P (2019). Strategic choices in polygamous households: Theory and evidence from senegal. The Review of Economic Studies.

[CR69] Runganga AO, Sundby J, Aggleton P (2001). Culture, identity and reproductive failure in Zimbabwe. Sexualities.

[CR70] Rutstein, S. O., Shah, I. H. (2004). Infecundity infertility and childlessness in developing countries. DHS Comparative Reports. Retrieved from: https://dhsprogram.com/pubs/pdf/CR9/CR9.pdf.

[CR71] Schoumaker B (2017). Measuring male fertility rates in developing countries with demographic and health surveys: An assessment of three methods. Demographic Research.

[CR72] Schoumaker B (2019). Male fertility around the world and over time: How different is it from female fertility. Population and Development Review.

[CR73] Sengupta P, Nwagha U, Dutta S, Krajewska-Kulak E, Izuka E (2017). Evidence for decreasing sperm count in African population from 1965 to 2015. African Health Sciences.

[CR74] Shapiro D, Gebreselassie T (2014). Marriage in sub-Saharan Africa: Trends, determinants, and consequences. Population Research and Policy Review.

[CR75] Smits J, Permanyer I (2019). The subnational human development database. Scientific Data.

[CR76] Snijders, T. A., Bosker, R. J. (2011). *Multilevel analysis: An introduction to basic and advanced multilevel modeling*. Sage Publications.

[CR77] Steinbrook R (2008). The aids epidemic- a progress report from Mexico City. New England Journal of Medicine.

[CR78] Surkyn J, Lesthaeghe R (2004). Value orientations and the second demographic transition (SDT) in Northern, Western and Southern Europe: An update. Demographic Research.

[CR79] Tertilt M (2005). Polygyny, fertility, and savings. Journal of Political Economy.

[CR80] The World Bank (2018). Fertility rate, total (births per woman) - sub-Saharan Africa. Retrieved from: https://data.worldbank.org/indicator/SP.DYN.TFRT.IN?locations=ZG.

[CR81] Thornton A (2013). Reading history sideways: The fallacy and enduring impact of the developmental paradigm on family life.

[CR82] Tsui AO, Brown W, Li Q (2017). Contraceptive practice in sub-Saharan Africa. Population and Development Review.

[CR83] UNDP (2015). Statistics of the Human Development Report. UNDP Human Development Report Office, New York. Retrieved from: http://hdr.undp.org/en/statistics/.

[CR84] United Nations (2000). *Demographic yearbook. Historical supplement 1948–1997*. United Nations.

[CR85] United Nations Desa/Population Division (2017a). Annual population by five-year age groups - female. De facto population as of 1 July of the year indicated classified by five-year age groups (0-4, 5-9, 10-14, ..., 95-99, 100+) [Data set]. Retrieved from: https://esa.un.org/unpd/wpp/DVD/Files/1_Indicators%20(Standard)/EXCEL_FILES/1_Population/WPP2017_POP_F15_3_ANNUAL_POPULATION_BY_AGE_FEMALE.xlsx.

[CR86] United Nations Desa/Population Division (2017b). Annual population by five-year age groups - male. De facto population as of 1 July of the year indicated classified by five-year age groups (0-4, 5-9, 10-14, ..., 95-99, 100+) [Data set]. Retrieved from: https://esa.un.org/unpd/wpp/DVD/Files/1_Indicators%20(Standard)/EXCEL_FILES/1_Population/WPP2017_POP_F15_2_ANNUAL_POPULATION_BY_AGE_MALE.xlsx.

[CR87] United Nations Development Programme [UNDP] (2019). Human development report: Technical notes. United Nations Development Programme (UNDP) Human Development Report Office, New York. Retrieved from: http://hdr.undp.org/sites/default/files/hdr2019_technical_notes.pdf.

[CR88] United States Agency for International Development (2018). Regional HIV prevalence among young men and women, 15–24 [Data set]. Retrieved from: https://www.statcompiler.com/en/.

[CR91] Westoff, C. F. (2003). Trends in marriage and early childbearing in developing countries. DHS Comparative Reports. Retrieved from: https://www.dhsprogram.com/pubs/pdf/CR5/CR5.pdf.

[CR92] White IR, Royston P, Wood AM (2011). Multiple imputation using chained equations: Issues and guidance for practice. Statistics in Medicine.

[CR93] Wilson C (2011). Understanding global demographic convergence since 1950. Population and Development Review.

[CR94] World Economic Forum (2018). The global gender gap report 2018. Retrieved from: http://www3.weforum.org/docs/WEF_GGGR_2018.pdf.

[CR95] World Health Organization (2014). Sexual and reproductive health: Infertility is a global public health issue. Retrieved from: http://www.who.int/reproductivehealth/topics/infertility/.

