# Supplementary material for: Childlessness and Development in Sub-Saharan Africa: Is There Evidence for a U-shaped Pattern?
Source: Eur J Popul. 2022 Mar 10;38(3):319–52. doi: 10.1007/s10680-022-09608-5 (PMC9363553; doi:10.1007/s10680-022-09608-5)
Supplement: Supplementary file 1 — (pdf 11764 KB) [file 10680_2022_9608_MOESM1_ESM.pdf]

Supplementary Information for

Childlessness and Development in sub-Saharan Africa: Is There Evidence for a U-shaped Pattern?

European Journal of Population

[Author information can and will be included in the final version but is excluded here for the purpose of blind review.]

List of Tables

S1 Countries under analysis and original and selected sample sizes . . . 1
S2 Goodness-of-fit measures and ANOVA p-values for national-level bivariate models for childlessness and SHIHD 19
S3 Goodness-of-fit measures and ANOVA p-values for subnational-level bivariate models for childlessness and SHIHD 19
S4 Goodness-of-fit measures for subnational-level multivariate models for childlessness 20
S5 Goodness-of-fit measures and χ^2 p-values for basic univariate models 21
S6 Random effects for subnational-level multivariate total models for childlessness (M5) 22
S7 Regression coefficients of the subnational regional-level bivariate models estimating childlessness types from development components for men and women 24

List of Figures

S1 Overview of male and female DHS surveys (black squares indicate survey years) 2
S2 Distribution of male and female childlessness and age at first birth across age 3
S3 Distribution of male and female ages per country 4
S4 SHDI trends per component by country and subnational region 5
S5 Trend of the HIHD and its components by country 12
S6 Scatter plot of sample sizes and childlessness levels (each point represents a unique subnational region-year combination) . . . 13
S7 Aggregation plot for missingness in male data 14
S8 Aggregation plot for missingness in female data 14
S9 Correlation between independent variables in male data 17
S10 Correlation between independent variables in female data . . . 17
S11 National trends in overall, involuntary, voluntary and circumstantial childlessness by country and gender 18
S12 Marginal effect on childlessness of each independent variable from full control model M5 23

Supplementary Information A Countries and Original and Selected Sample Sizes

Table S1: Countries under analysis and original and selected sample sizes

Country	ISO3	Men		Women	
		Selected	Original	Selected	Original
Angola	AGO	1001	5684	1721	25940
Benin	BEN	6033	22340	9804	62031
Burkina Faso	BFA	4055	15398	8881	58102
Burundi	BDI	3072	11832	4900	35777
Cameroon	CMR	3830	15847	5095	35454
Central African Republic	CAF	415	1729	886	5884
Chad	TCD	2183	9455	4489	31258
Comoros	COM	726	2962	1179	8379
Congo	COG	2040	8291	4806	30283
Congo Democratic Republic	COD	3447	13413	4255	28822
Cote d'Ivoire	CIV	2093	8573	4637	30885
Ethiopia	ETH	11953	48126	11510	77318
Gabon	GAB	1733	5654	1611	8422
Gambia	GMB	728	3821	1173	10233
Ghana	GHA	4726	16819	7195	39046
Guinea	GIN	2226	8936	3260	23849
Kenya	KEN	8734	38424	17482	106762
Lesotho	LSO	2065	9045	3687	21340
Liberia	LBR	2121	10127	5613	34196
Madagascar	MDG	3035	11018	11147	65513
Malawi	MWI	4445	22157	12578	87012
Mali	MLI	5986	19103	10861	69037
Mozambique	MOZ	3778	14553	11703	60087
Namibia	NAM	2489	11350	5303	31998
Niger	NER	3982	12589	4876	34463
Nigeria	NGA	8389	35191	18151	103112
Rwanda	RWA	6452	27518	11243	72931
Sao Tome and Principe	STP	600	2296	506	2615
Senegal	SEN	9076	32041	18130	127331
Sierra Leone	SLE	3008	10542	4973	32533
South Africa	ZAF	1044	3618	3844	20249
Sudan	SDN			1170	5860
Swaziland	SWZ	538	4156	825	4987
Tanzania	TZA	3427	16588	18938	113298
Togo	TGO	2011	8295	4093	26083
Uganda	UGA	2797	14092	14408	85954
Zambia	ZMB	5764	25267	6560	46296
Zimbabwe	ZWE	6746	36197	8021	54224

Supplementary Information B Overview of DHS Surveys

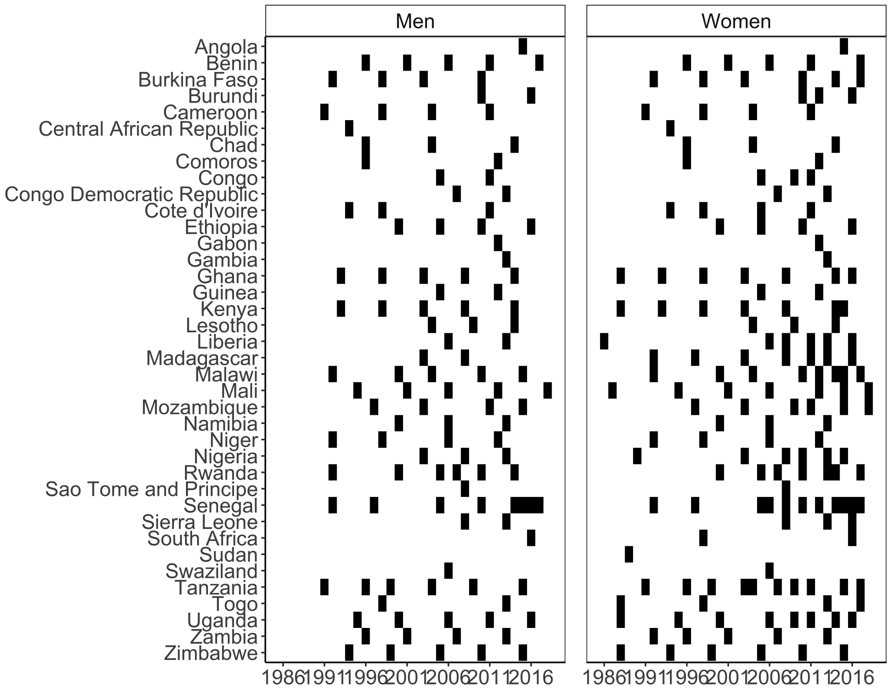

Figure S1: Overview of male and female DHS surveys (black squares indicate survey years)

Supplementary Information C Distribution of Childlessness and Age at First Birth

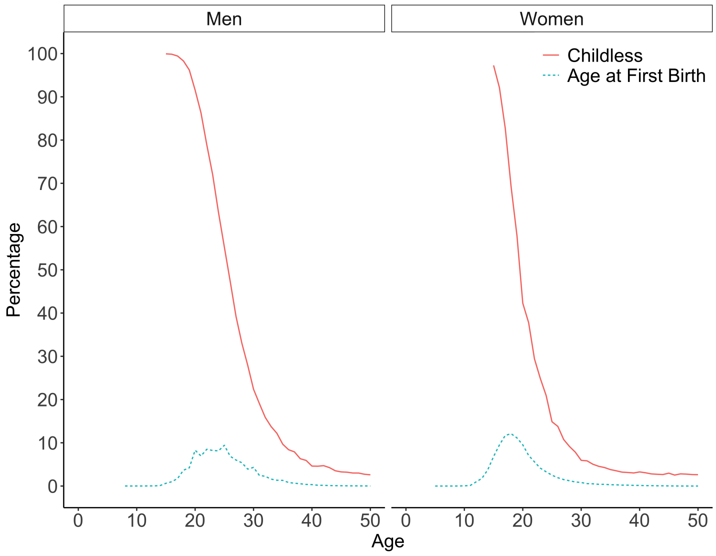

Figure S2: Distribution of male and female childlessness and age at first birth across age

Supplementary Information D Age Distributions per Country

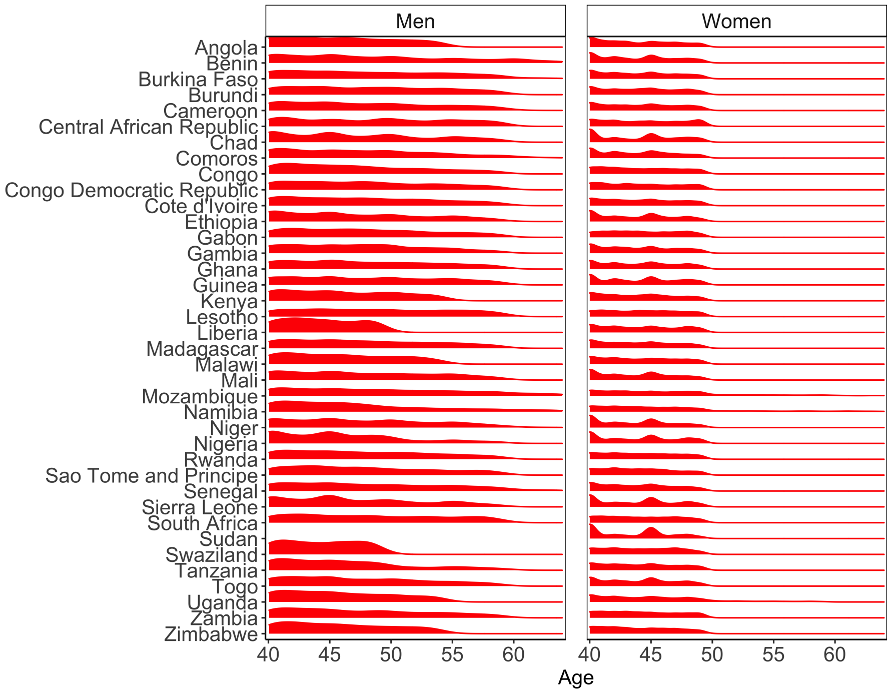

Figure S3: Distribution of male and female ages per country

Supplementary Information E Trends of SHIHD

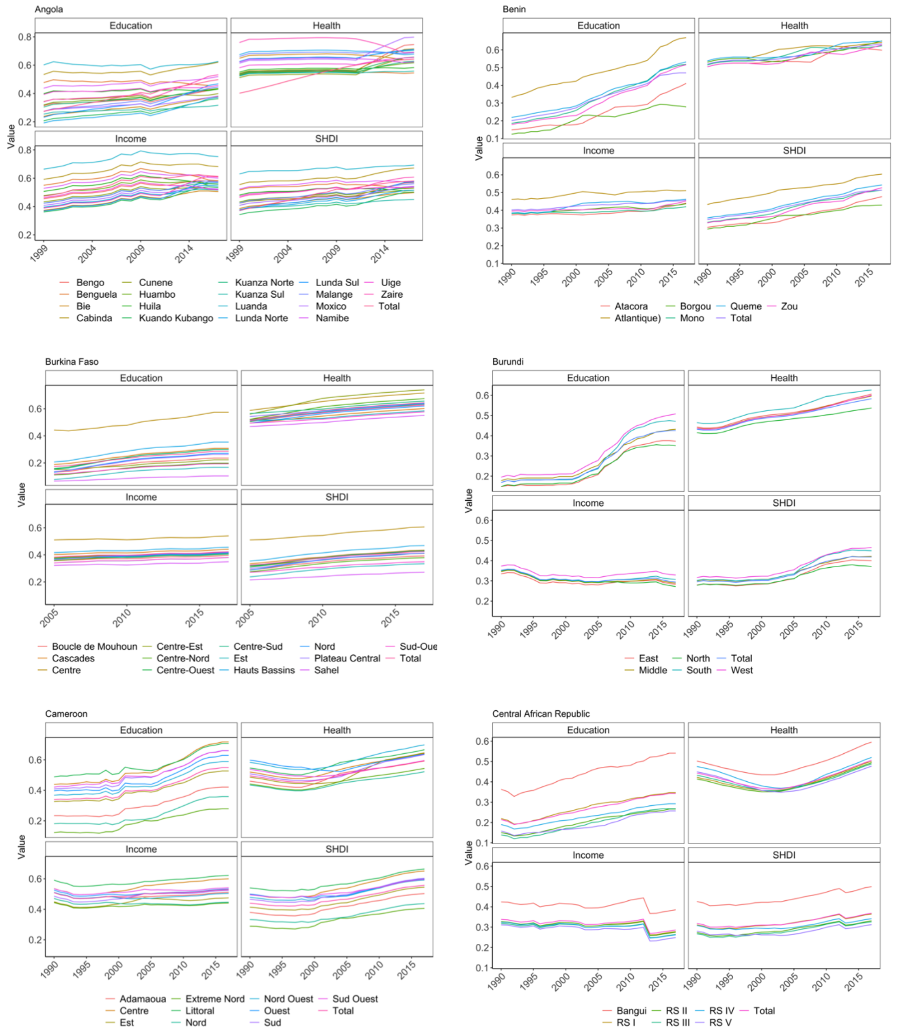

Figure S4: SHDI trends per component by country and subnational region

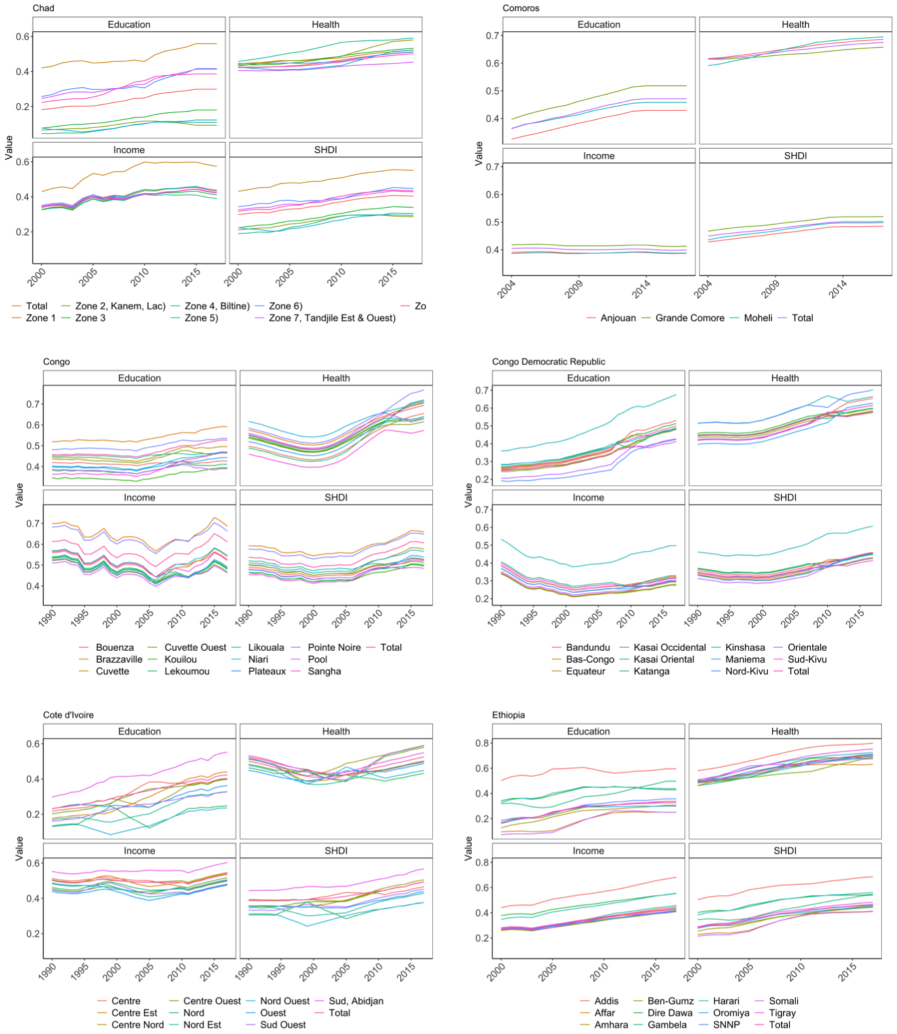

Figure S4 (cont.): SHDI trends per component by country and subnational region

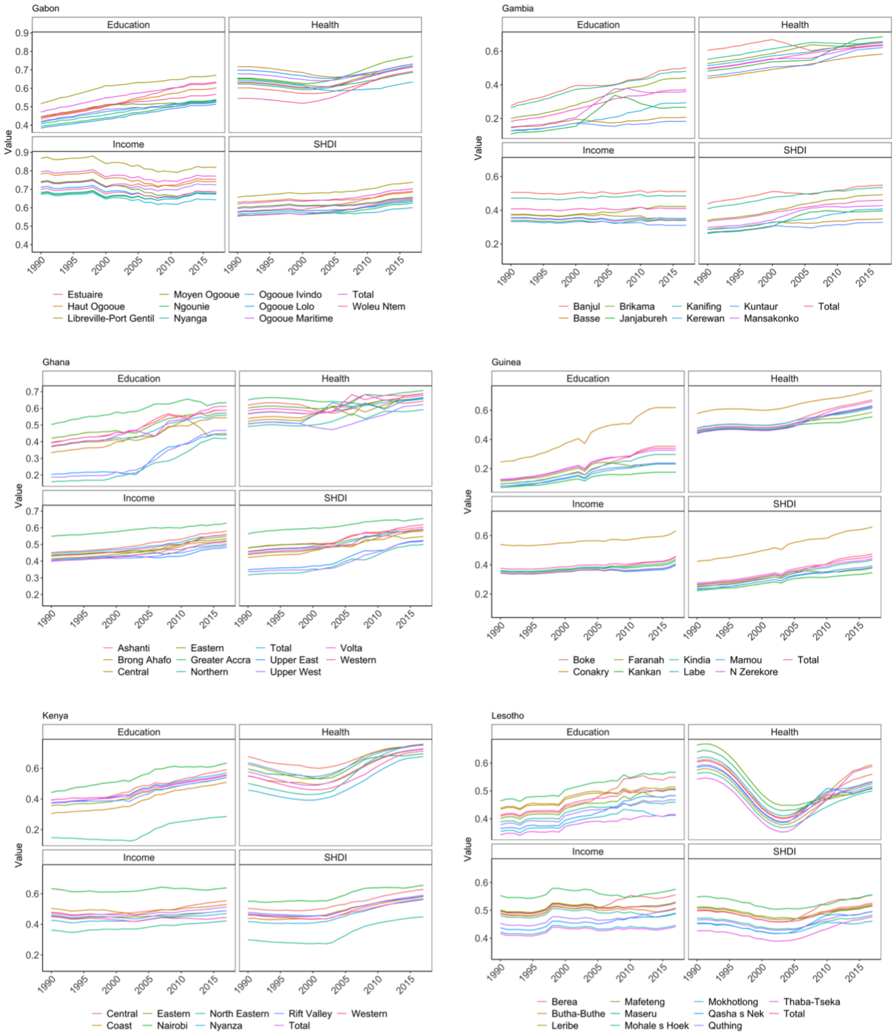

Figure S4 (cont.): SHDI trends per component by country and subnational region

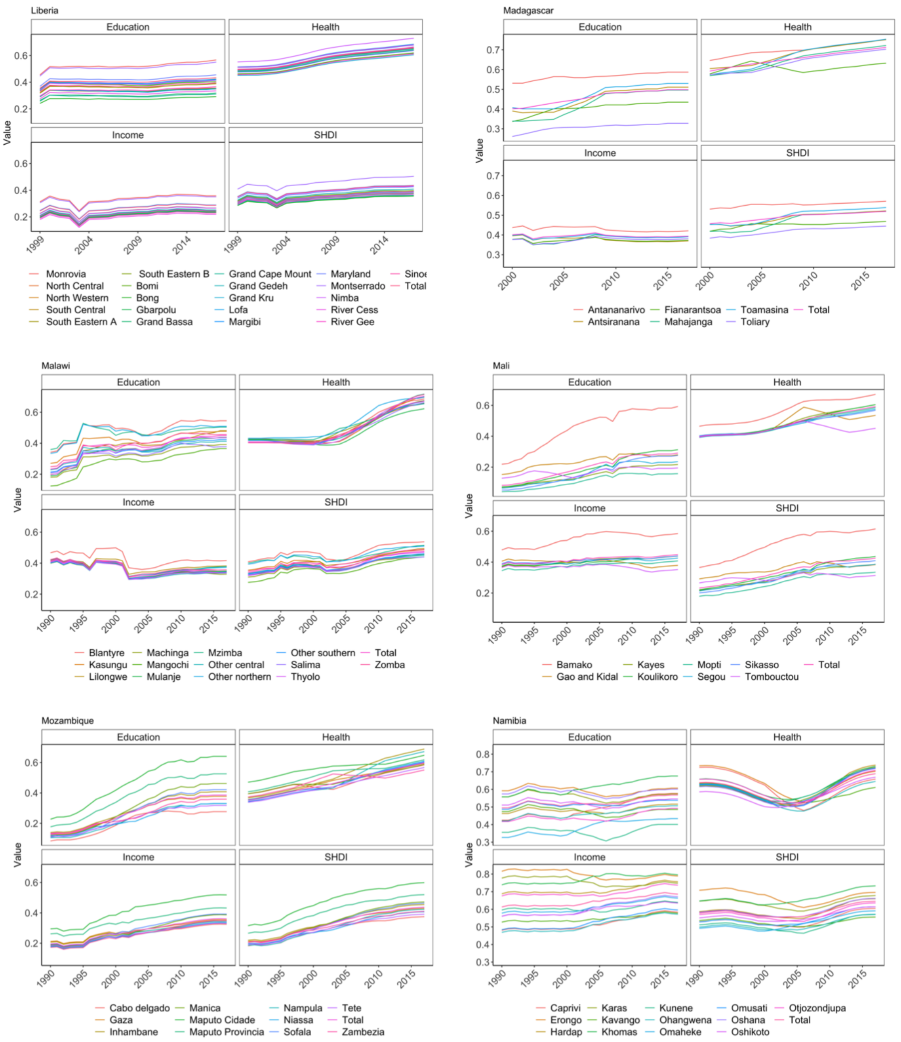

Figure S4 (cont.): SHDI trends per component by country and subnational region

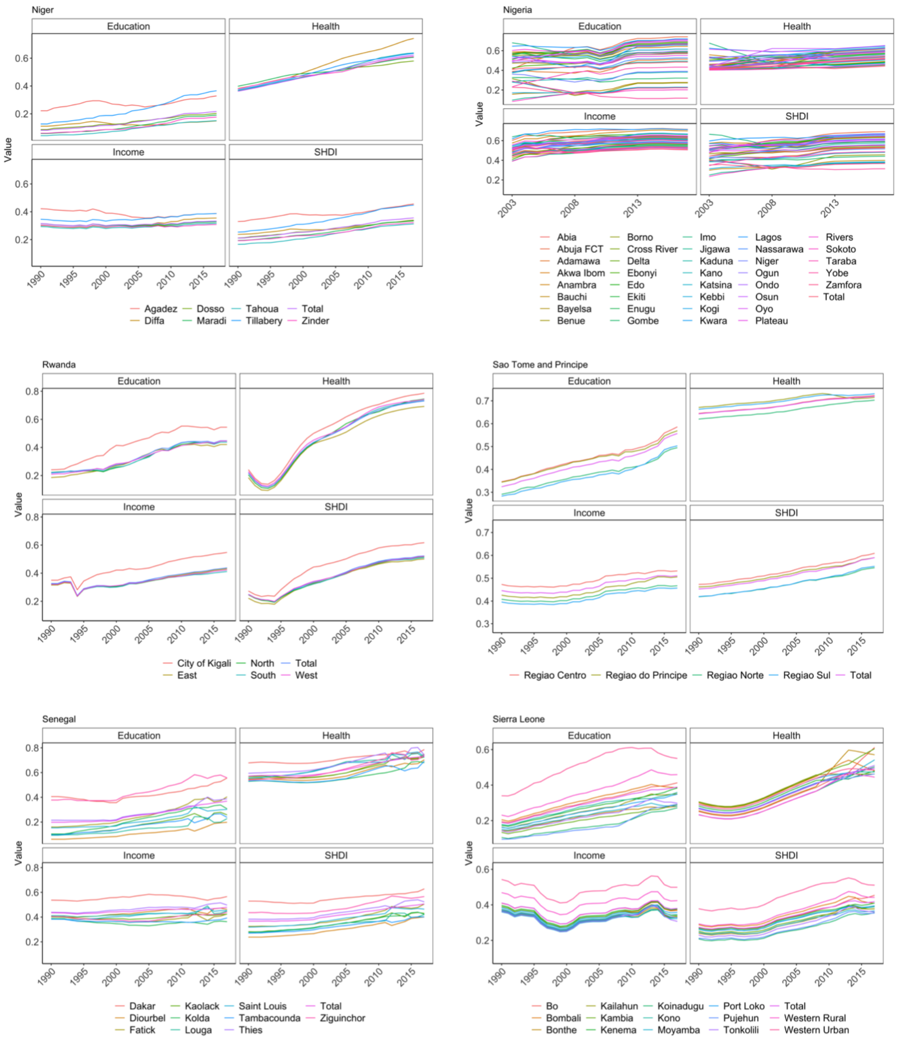

Figure S4 (cont.): SHDI trends per component by country and subnational region

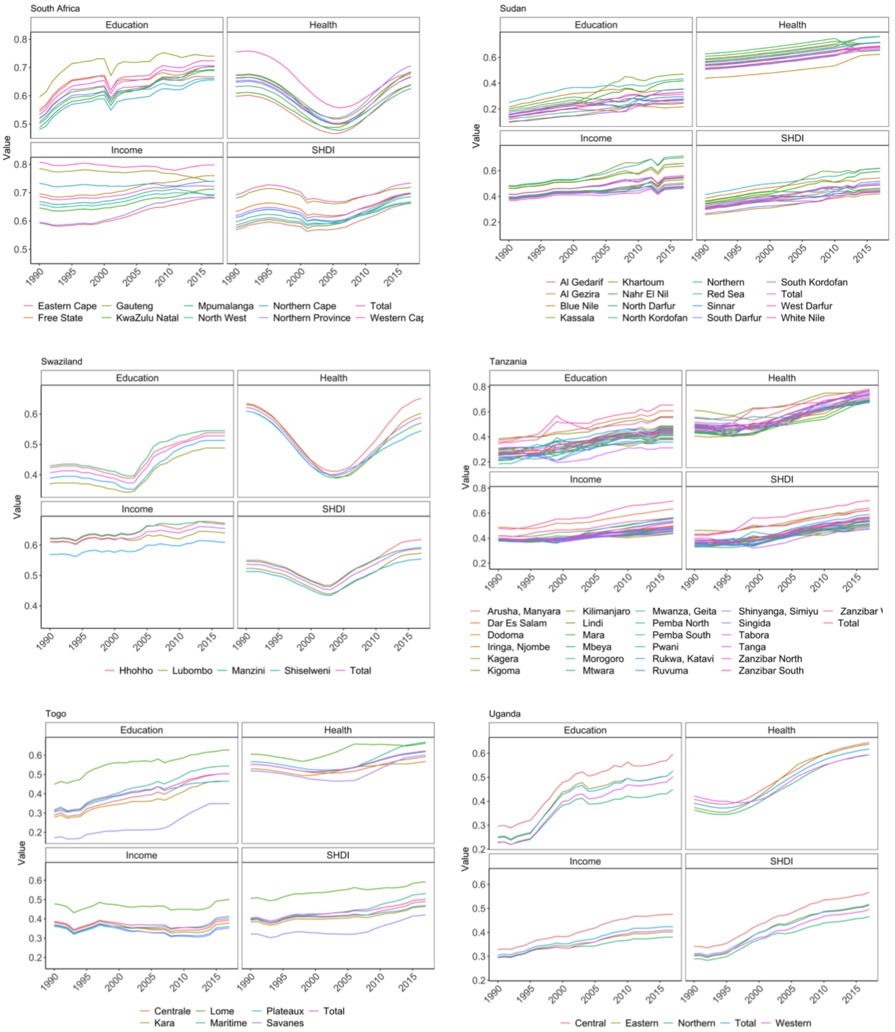

Figure S4 (cont.): SHDI trends per component by country and subnational region

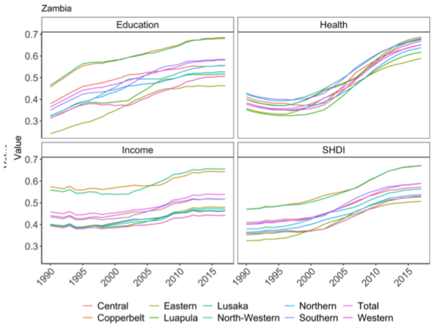

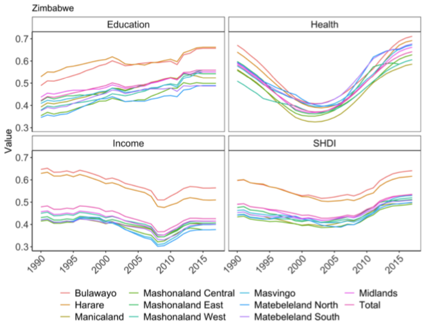

Figure S4 (cont.): SHDI trends per component by country and subnational region

Supplementary Information F Trends of HIHD and components for sub-Saharan Africa

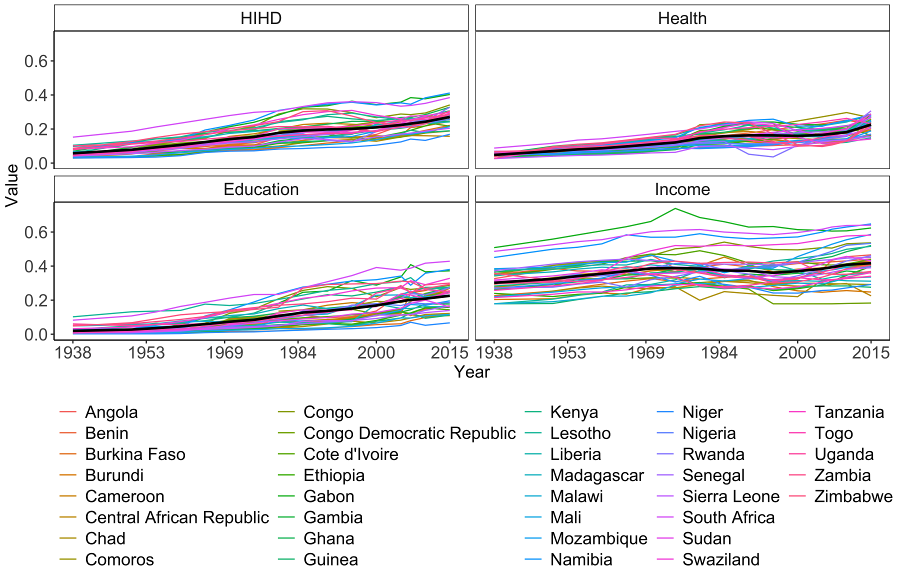

Figure S5: Trend of the HIHD and its components by country (black line shows unweighted average across sub-Saharan African countries)

Supplementary Information G Childlessness and Sample Size

Theoretically, aggregating individual-level childlessness to the percentage of childlessness in subnational region-year combinations may lead to many zeros in our dependent variable in the case of small sample sizes for region-year combinations. Figure S6 shows a scatter plot of sample sizes against childlessness percentages for unique subnational region-year combinations, and shows that there are relatively few (16% for women and 18% for men) zeros in our dependent variable and that these are not necessarily due to aggregation of data from small samples, as the zeros range between sample sizes of 1 and 265.

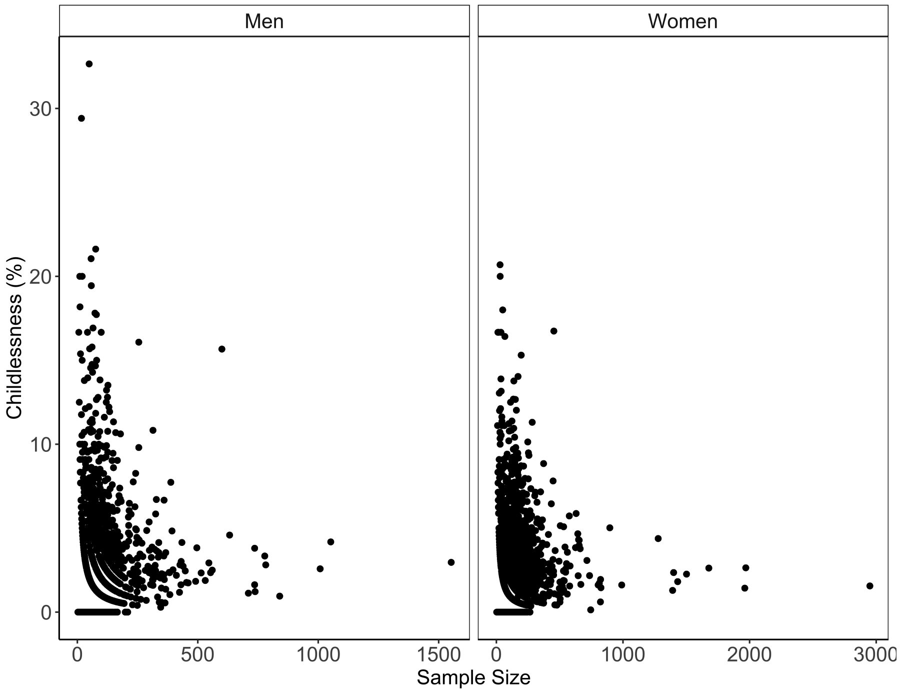

Figure S6: Scatter plot of sample sizes and childlessness levels (each point represents a unique subnational region-year combination)

Supplementary Information H Missingness in the Data

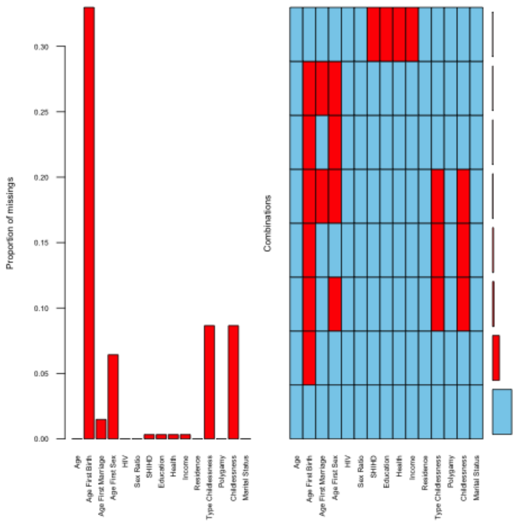

Figure S7: Aggregation plot for missingness in male data

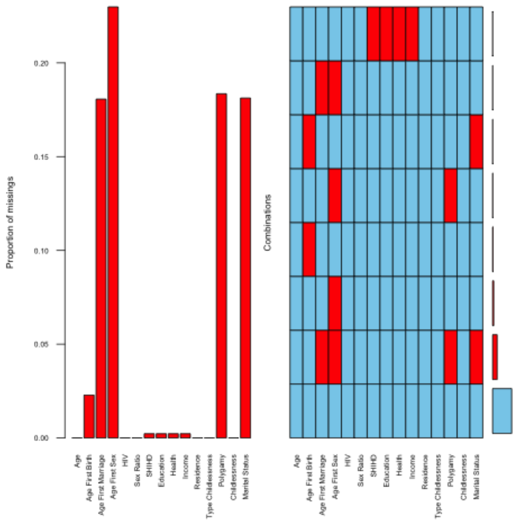

Figure S8: Aggregation plot for missingness in female data

Supplementary Information I Computation of Orthogonal Polynomials

For a vector $\underline{x}$ of length n, we can generate monic orthogonal polynomials of degree d, $\underline{P}_0(\underline{x}), \underline{P}_1(\underline{x}), \ldots, \underline{P}_d(\underline{x})$, as follows (pp.343-4 Kennedy and Gentle, 1980):

1. Center $\underline{x}$ by its mean as $\underline{x} = \underline{x} - \bar{\underline{x}}$.
2. Set $\underline{P}_{-1}(\underline{x}) = 0, \underline{P}_0(\underline{x}) = 1, \underline{P}_1(\underline{x}) = \underline{x}$ (notice the orthogonality here).
3. For $i = 0, 1, \ldots, d$, define $l_i = \langle \underline{P}_i(\underline{x}), \underline{P}_i(\underline{x}) \rangle$ and $\alpha_i = \frac{\langle \underline{P}_i^2(\underline{x}), \underline{x} \rangle}{l_i}$ and for $i = 1, 2, \ldots, d$ define $\beta_i = \frac{l_i}{l_{i-1}}$
4. For $i = 2, 3, \ldots, d$ derive $\underline{P}_i(\underline{x})$ as $\underline{P}_i(\underline{x}) = (x - \alpha_{i-1})\underline{P}_{i-1}(\underline{x}) - \beta_{i-1}\underline{P}_{i-2}(\underline{x})$.
5. For $i = 0, 1, \ldots, d$ scale $\underline{P}_i(\underline{x})$: $\underline{P}_i(\underline{x}) = \frac{\underline{P}_i(\underline{x})}{\sqrt{l_i}}$.
6. Use $\underline{P}_1(\underline{x}), \underline{P}_2(\underline{x}), \ldots, \underline{P}_d(\underline{x})$ as regressors.

Supplementary Information J Pooling Strategy for Hierarchical Regression Results after Multiple Imputation

We impute our data M = 36 and M = 29 times respectively for men and women, approximately equalling the percentage of incomplete observations as recommended by Bodner (2008); White et al. (2011). We run each of the aforementioned models on each of these M imputed data sets and we pool the results by taking the mean of the resulting M k-length vectors of coefficients $\underline{\pi}_1^*, \underline{\pi}_2^*, \ldots, \underline{\pi}_M^*$ as

$$\hat{\underline{\pi}}^* = \frac{1}{M}\sum_{i=1}^{M} \underline{\pi}_i^* \tag{7}$$

and by computing the pooled variance as

$$\begin{aligned}\hat{\underline{V}}^* &= \hat{\underline{W}}^* + \frac{M+1}{M}\hat{\underline{B}}^* \\ &= \frac{1}{M}\sum_{i=1}^{M}\underline{U}_i^* + \frac{M+1}{M}\frac{1}{M-1}\sum_{i=1}^{M}(\underline{\pi}_i^* - \hat{\underline{\pi}}^*)^2\end{aligned} \tag{8}$$

where $\hat{\underline{W}}^*$ is the average within-imputation variance; $\underline{U}_i^*$ is the within-imputation variance (i.e. the variance of π that would be observed if the imputed data would be the true observed data); and $\hat{\underline{B}}^*$ is the between-imputation variance. Note that $\hat{\underline{V}}^*, \hat{\underline{W}}^*$ and $\hat{\underline{B}}^*$ are vectors of length k. We further adjust the distribution of $\hat{\underline{\pi}}^*$ for small sample sizes as recommended by Barnard and Rubin (1999) by basing the inference for the coefficient of variable $j = 1, \ldots, k$ on a t-distribution rather than asymptotic normal distribution using

$$\tilde{\nu}_{M,j} = \frac{\frac{M-1}{\gamma_{M,j}^2}\nu_{obs,j}}{\frac{M-1}{\gamma_{M,j}^2} + \nu_{obs,j}} \tag{9}$$

degrees of freedom, where

$$\nu_{obs,j} = \frac{\nu_{comp}+1}{\nu_{comp}+3}\nu_{comp}(1-\hat{\gamma}_{M,j}) \tag{10}$$

denotes the estimated observed-data degrees of freedom with $\nu_{comp} = n-k-1$ the complete-data degrees of freedom and $\hat{\gamma}_{M,j} = \frac{M}{M+1}\frac{\hat{B_j}^*}{\hat{V_j}^*}$ the estimated proportion of missing information for the jth variable.

Supplementary Information K Correlation Between Independent Variables

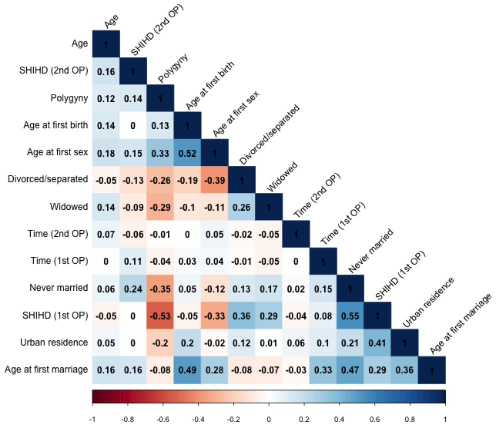

Figure S9: Correlation between independent variables in male data

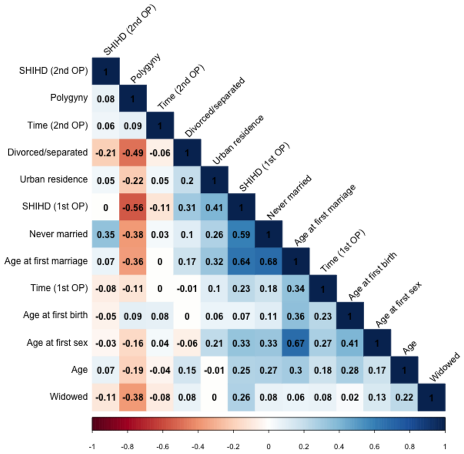

Figure S10: Correlation between independent variables in female data

Supplementary Information L Childlessness Trends by Country, Gender and Type

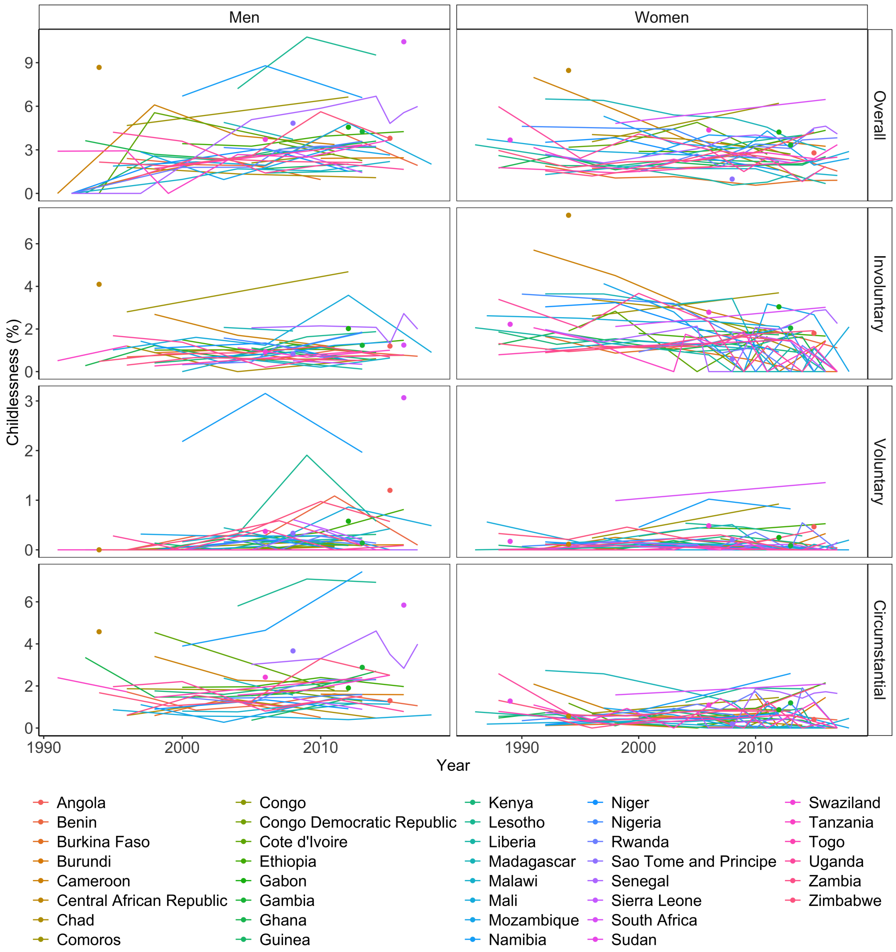

Figure S11: National trends in overall, involuntary, voluntary and circumstantial childlessness by country and gender

Supplementary Information M Goodness-of-Fit Measures for Sub-National-Level Models

Table S2 shows the linear and quadratic goodness-of-fit measures for the national-level bivariate regression with the percentage of childlessness as the dependent variable and the second-order orthogonal polynomials of the SHIHD term as the independent variables. The p-values shown in the table refer to the p-values resulting from ANOVA used to compare linear models against their quadratic counterparts, with a significance threshold of 0.001.

Table S2: Goodness-of-fit measures and ANOVA p-values for national-level bivariate models for childlessness and SHIHD

Component	Fit	Men				Women			
		AIC	BIC	RMSE	P-value	AIC	BIC	RMSE	P-value
SHIHD	Linear	445.484	453.530	1.851		575.401	584.700	1.373	
	Quadratic	442.681	453.409	1.810	0.031	574.411	586.810	1.361	0.087
Education	Linear	449.736	457.782	1.888		575.754	585.053	1.375	
	Quadratic	442.240	452.968	1.807	0.002	573.994	586.394	1.359	0.055
life expectancy	Linear	451.978	460.024	1.907		578.914	588.214	1.388	
	Quadratic	450.324	461.053	1.875	0.060	576.542	588.942	1.369	0.039
Income	Linear	449.427	457.473	1.885		566.129	575.428	1.335	
	Quadratic	450.172	460.901	1.874	0.271	567.828	580.228	1.334	0.588

Table S3 shows the linear and quadratic goodness-of-fit measures for the subnational-level bivariate regression with the percentage of childlessness as the dependent variable and orthogonal polynomial terms of SHIHD as the independent variable(s). The p-values shown in the table refer to the p-values resulting from ANOVA used to compare linear models against their quadratic counterparts.

Table S3: Goodness-of-fit measures and ANOVA p-values for subnational-level bivariate models for childlessness and SHIHD

Component	Fit	Men				Women			
		AIC	BIC	RMSE	P-value	AIC	BIC	RMSE	P-value
SHIHD	Linear	5886.668	5901.688	3.471		8325.861	8342.255	2.625	
	Quadratic	5879.635	5899.662	3.457	0.003	8316.169	8338.027	2.616	0.001
Education	Linear	5908.421	5923.441	3.505		8332.759	8349.152	2.630	
	Quadratic	5892.644	5912.670	3.477	0.000	8319.850	8341.708	2.619	0.000
life expectancy	Linear	5925.342	5940.362	3.532		8357.599	8373.992	2.649	
	Quadratic	5923.063	5943.089	3.525	0.039	8346.887	8368.745	2.639	0.000
Income	Linear	5927.555	5942.575	3.536		8275.367	8291.761	2.587	
	Quadratic	5929.180	5949.207	3.535	0.541	8276.987	8298.845	2.587	0.538

Table S4 shows the linear and quadratic goodness-of-fit measures for the subnational-level multivariate regression with the percentage of childlessness as the dependent variable.

Table S4: Goodness-of-fit measures for subnational-level multivariate models for childlessness

Model	Fit	Men				Women			
		AIC	BIC	RMSE	P-value	AIC	BIC	RMSE	P-value
SHIHD	Linear	-4927.427	-4891.721	0.03		-8010.891	-7972.623	0.023	
	Quadratic	-4916.195	-4854.985	0.029	0.002	-8097.594	-8031.992	0.022	0.000
Age & Residence	Linear	-4907.64	-4861.732	0.03		-7997.235	-7948.034	0.023	
	Quadratic	-4896.052	-4824.641	0.029	0.003	-8081.931	-8005.396	0.022	0.000
Marriage & Postponement	Linear	-5191.312	-5125.001	0.026		-7974.96	-7903.892	0.023	
	Quadratic	-5175.319	-5083.503	0.026	0.903	-8060.544	-7962.141	0.022	0.000
Polygyny, HIV & Sex Ratio	Linear	-4914.396	-4863.388	0.029		-8002.168	-7947.5	0.023	
	Quadratic	-4900.304	-4823.792	0.029	0.089	-8089.681	-8007.679	0.022	0.000
Total	Linear	-5141.966	-5050.151	0.026		-7945.014	-7846.612	0.022	
	Quadratic	-5125.666	-5008.346	0.026	0.992	-8018.6	-7892.864	0.022	0.000

Supplementary Information N Model Comparison for Basic Models

Table S5 shows the goodness-of-fit measures for the subnational-level univariate basic models used to build the hierarchical model. The *intercept* model predicts childlessness with only a fixed intercept; the *randomIntercept* model predicts childlessness with an intercept allowed to vary across countries; the *shihdRI* model adds a first-order SHIHD orthogonal polynomial term to the *randomIntercept* model; the *shihdRS* model additionally allows the effect of a first-order SHIHD orthogonal polynomial term to vary across countries; and the *shihdQ* model is the same as *shihdRS* model but then with second-order orthogonal polynomials of the SHIHD term. As the random effects specifications for the models in Table S5 differ, we have compared model fits between two models A and B by executing a χ^2-test with two degrees of freedom and as parameter the difference between the $-2{\cdot}$log-likelihood of models A and B. As adding the second-order orthogonal polynomial term for SHIHD does not significantly improve the model for men, we fit our hierarchical models linearly for men.

Table S5: Goodness-of-fit measures and χ^2 p-values for basic univariate models

Model	Men				Women			
	AIC	BIC	RMSE	P-value	AIC	BIC	RMSE	P-value
intercept	-4572.314	-4562.112	0.037		-7733.605	-7722.671	0.026	
randomIntercept	-4825.503	-4810.200	0.031	0.000	-7922.381	-7905.981	0.024	0.000
shihdRI	-4905.384	-4884.981	0.030	0.000	-7934.131	-7912.264	0.024	0.001
shihdRS	-4933.597	-4902.992	0.030	0.000	-8004.982	-7972.182	0.023	0.000
shihdQ	-4927.688	-4876.680	0.029	0.351	-8103.317	-8048.649	0.022	0.000

Supplementary Information O Random Effects for Hierarchical Models

The random effects of the total male and female models (M5) in Table S6 show that while the baseline childlessness level hardly varies across countries, the effects of the linear (and, in the case of women, quadratic) SHIHD terms vary quite substantially across countries. Further, there is a positive correlation between the baseline childlessness level and the effect of the first- and, in the case of females, second-order orthogonal polynomial terms, suggesting that the effect of SHIHD is larger for countries where the baseline childlessness level is higher. Finally, there is a positive correlation between the effects of the linear and quadratic terms of SHIHD among women.

Table S6: Random effects for subnational-level multivariate total models for childlessness (M5)

Group	Variable 1	Variable 2	Men			Women		
			Var.	St. Dev.	Corr.	Var.	St. Dev.	Corr.
Country	Intercept		0.000	0.009		0.000	0.015	
Country	SHIHD (1st order polynomial)		0.046	0.213		0.556	0.746	
Country	SHIHD (2nd order polynomial)					0.156	0.395	
Country	Intercept	SHIHD (1st order polynomial)	0.001	0.555	0.702	0.003	0.228	0.170
Country	Intercept	SHIHD (2nd order polynomial)				0.003	0.472	0.441
Country	SHIHD (1st order polynomial)	SHIHD (2nd order polynomial)				0.194	0.659	0.735
Residual			0.001	0.027		0.000	0.022	

Supplementary Information P Marginal Effects of Independent Variables on Childlessness

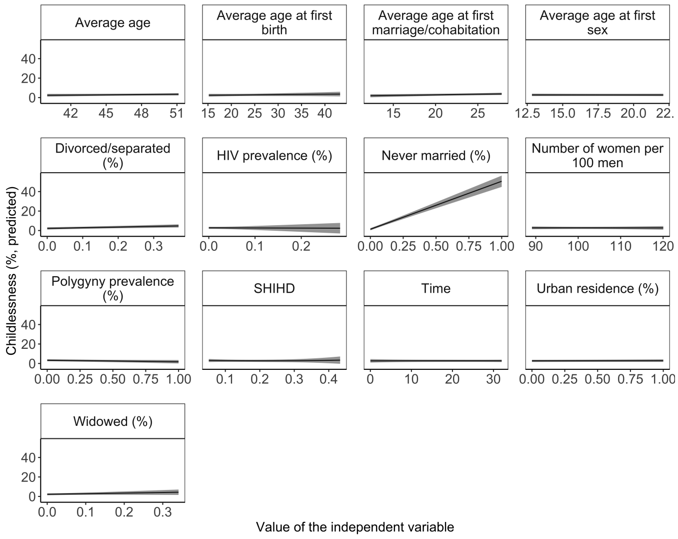

(a) Marginal effect on childlessness of each independent variable from full control model M5 (men, linear)

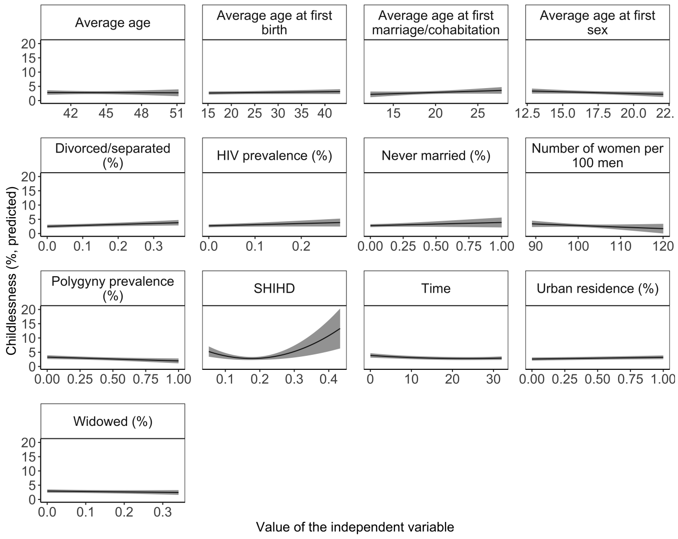

(b) Marginal effect on childlessness of each independent variable from full control model M5 (women, quadratic)

Figure S12: Marginal effect on childlessness of each independent variable from full control model M5

Supplementary Information Q Bivariate Regression Results for Types of Childlessness and development Components

Table S7: Regression coefficients of the subnational regional-level bivariate models estimating childlessness types from development components for men and women

Component	Coefficient	Men (N = 1,104)			Women (N = 1,745)		
		Involuntary	Voluntary	Circumstantial	Involuntary	Voluntary	Circumstantial
Education	Intercept	0.011***	0.003***	0.021***	0.015***	0.001***	0.006***
		(0.001)	(0)	(0.001)	(0)	(0)	(0)
	poly(Education, 1)	0.031+	0.098***	0.296***	-0.073***	0.033***	0.113***
		(0.017)	(0.010)	(0.025)	(0.019)	(0.004)	(0.011)
	AIC	-5849.414	-6928.039	-4993.595	-8905.145	-14083.404	-10681.836
	BIC	-5834.394	-6913.018	-4978.575	-8888.751	-14067.010	-10665.442
	RMSE	0.017	0.010	0.025	0.019	0.004	0.011
Income	Intercept	0.011***	0.003***	0.021***	0.015***	0.001***	0.006***
		(0.001)	(0)	(0.001)	(0)	(0)	(0)
	poly(Income, 1)	0.055**	0.107***	0.237***	0.071***	0.040***	0.107***
		(0.017)	(0.010)	(0.026)	(0.019)	(0.004)	(0.011)
	AIC	-5856.538	-6945.046	-4945.349	-8904.525	-14112.250	-10671.220
	BIC	-5841.518	-6930.026	-4930.329	-8888.131	-14095.856	-10654.827
	RMSE	0.017	0.010	0.026	0.019	0.004	0.011
life expectancy	Intercept	0.011***	0.003***	0.021***	0.015***	0.001***	0.006***
		(0.001)	(0)	(0.001)	(0)	(0)	(0)
	poly(life expectancy, 1)	0.027	0.084***	0.288***	-0.052**	0.025***	0.074***
		(0.017)	(0.011)	(0.025)	(0.019)	(0.004)	(0.012)
	AIC	-5848.650	-6905.219	-4986.693	-8897.880	-14058.215	-10625.295
	BIC	-5833.630	-6890.199	-4971.673	-8881.486	-14041.822	-10608.901
	RMSE	0.017	0.011	0.025	0.019	0.004	0.012

Note: $^{+}p < 0.1$; $^{*}p < 0.05$; $^{**}p < 0.01$; $^{***}p < 0.001$
